# Attenuation of dermal wounds through topical application of ointment containing phenol enriched fraction of *Caesalpinia mimosoides* Lam.

**DOI:** 10.3389/fphar.2022.1025848

**Published:** 2022-10-13

**Authors:** Pradeep Bhat, Vinayak Upadhya, Ganesh R. Hegde, Harsha V. Hegde, Subarna Roy

**Affiliations:** ^1^ National Institute of Traditional Medicine, Indian Council of Medical Research, Belagavi, India; ^2^ Department of Studies in Botany, Karnatak University, Dharwad, India; ^3^ Department of Forest Products and Utilization, College of Forestry, University of Agricultural Sciences, Sirsi, India

**Keywords:** wound healing, traditional medicine, *Caesalpinia mimosoides*, Western Ghats, antimicrobial, antioxidant, anti-inflammatory

## Abstract

*Caesalpinia mimosoides* Lam. is one of the important medicinal plants used by the traditional healers of Uttara Kannada district, Karnataka (India) for treating wounds. In our previous study ethanol extract of the plant was evaluated for its wound healing activity. In continuation, the present study was aimed to evaluate the phenol enriched fraction (PEF) of ethanol extract for wound healing activity along with its antioxidant, anti-inflammatory and antimicrobial properties. The potent wound healing activity of PEF was evidenced by observation of increased rate of cell migration in L929, 3T3L1 and L6 cells (92.59 ± 1.53%, 98.42 ± 0.82% and 96.63 ± 0.61% respectively) at 7.81 μg/ml doses in assays carried out *in vitro*. Significantly enhanced rate of wound contraction (97.92 ± 0.41%), tensile strength (973.67 ± 4.43 g/mm^2^), hydroxyproline (31.31 ± 0.64 mg/g) and hexosamine (8.30 ± 0.47 mg/g) contents were observed on 15th post wounding day in 5% PEF treated animals. The enzymatic and non-enzymatic cellular antioxidants (superoxide dismutase, catalase and reduced glutathione) were upregulated (15.89 ± 0.17 U/mg, 48.30 ± 4.60 U/mg and 4.04 ± 0.12 μg/g respectively) with the administration of 5% PEF. The significant antimicrobial, antioxidant and anti-inflammatory activities support the positive correlation of PEF with its enhanced wound healing activity. PEF contains expressive amounts of total phenolic and total flavonoid contents (578.28 ± 2.30 mg GAE/g and 270.76 ± 2.52 mg QE/g). Of the various chemicals displayed in RP-UFLC-DAD analysis of PEF, gallic acid (68.08 μg/mg) and ethyl gallate (255.91 μg/mg) were predominant. The results indicate that PEF has great potential for the topical management of open wounds.

## 1 Introduction

Skin is the major organ of human body which acts as a protective barrier against external stimuli and harmful causative factors. Wound is an inevitable part of life and is defined as “the physical injuries that result in disruption or breaking of the skin that causes disturbance in the structural integrity and functional continuity of skin tissue” (Strodtbeck, 2001; Azis et al., 2017). Wounds are classified as open and closed wounds on the basis of principal cause of wound creation and as acute and chronic wounds based on physiology of wound healing (Schultz, 1999).

The healing of cutaneous injury is a complex biological and molecular process, composed of four overlapping and interdependent phases *viz.* hemostasis, inflammation, proliferation and remodeling. Each phase of the healing process occurs in a precise manner, involving the production and participation of growth factors, cytokines, chemokines and various other types of extracellular matrix components to restore damaged tissue (Carvalho et al., 2021). The human body is fortunately facilitated with a self wound healing mechanism. However, in crucial circumstances such as microbial infections, diabetic impediments and poor blood circulation due to vascular deficits, wound management becomes difficult and causes a significant impact on health care economy (Azis et al., 2017). Though it is very intricate process to provide an accurate epidemiological data on the wounds, a retrospective limited data analysis of the medicare beneficiaries confirmed that chronic non-healing wounds impacts on 8.2 million (15%) of the total medicare beneficiaries all over the world (Nussbaum et al., 2018). The medicare cost projections of all types of wounds ranged between $28.1and $96.8 billion. Among these, surgical wounds ($11.7 to $38.3 billion) and diabetic foot ulcers ($6.2 to $18.7 billion) were most expensive treatments, including the infection managements. The risk of morbidity, psychological and higher socio-economic impact on the patients make wounds a severe public health crisis (Carvalho et al., 2021).

There are many medications available in the market for the treatment of wounds. However, most of them focus only on preventing inflammation and the growth of microbes at the site of the injury, and do not induce the healing process. Allergic reactions and multiple drug resistance are the major problems that limit the usage of many synthetic drugs (Shao et al., 2019). The exogenous growth factors delivered at the wound site would mimic the natural microenvironment of tissue formation hastening healing. These procedures are not only expensive and inaccessible to the common people, but also many of these synthetic drugs cause considerable side effects (Brem and Tomic-Canic, 2007). Alternative therapies using natural products, especially the bioactive botanicals having traditional history of use, are often the major resource in drug development. These natural compounds isolated from traditional medicinal plants are believed to induce healing and regeneration of the lost tissue by interacting with various molecular targets through multiple mechanisms. It is estimated that approximately 1/4th of the globally prescribed drugs are of herbal origin and 11% of 433 essential drugs compiled by WHO are derived from plant species (Semwal et al., 2019). In recent years, use of new plant-based compounds used as wound-healing agents are increasing, since the bacterial and fungal pathogens are developing multiple drug resistance against many of the existing antibiotics (Weckesser et al., 2007). Further efforts are being made to elucidate the molecular mechanisms and signaling pathways of active phytocompounds involved in wound healing (Elloumi et al., 2022).

Among the bioactive phytocompounds, polyphenols are one of the most important groups of plant secondary metabolites often constituting an integral component of healthcare system. They are the subject of increasing scientific interest in the treatment of wound complications due to their effective antimicrobial, antioxidant and anti-inflammatory properties (Yadav et al., 2018; Guimarães et al., 2021). More than 8,000 different polyphenolic compounds have been identified in various plant species and their origin is from phenylalanine or a close precursor, shikimic acid (Pandey and Rizvi., 2009). They are classified into flavonoid and non-flavonoid compounds based on the number and binding patterns of phenolic carbon units. Flavonoids are subdivided into six main subclasses (flavones, isoflavones, flavonones, flavonols, flavan-3-ols and anthocyanidinidins) whereas, non-flavonoids are phenolic acids, tannins and lignins (Działo et al., 2016).

During extensive ethnobotanical explorations in Uttara Kannada district of Central Western Ghats, India, we found *Caesalpinia mimosoides* Lam., as one of the ethnomedicinal plants highly cited by the traditional practitioners to treat the wounds and skin ailments (Bhat et al., 2012, 2014). *C. mimosoides* belongs to the family Leguminosae is a straggling shrub covered with small spines throughout. It is native to South-east Asia and Indian subcontinent (Nayar et al., 2014). The tender leaves are consumed as vegetable and as an appetizer in Thailand (Chanwitheesuk et al., 2007). In Kerala state of India, the Mullu kuruma tribe uses this plant for the treatment of epilepsy (Silja et al., 2008). It has antimicrobial (Silja et al., 2008), anti-inflammatory (Yodsaoue et al., 2010), antioxidant (Chanwitheesuk et al., 2005) and anticancer (Daduang et al., 2011; Palasap et al., 2014) properties. Further, the quercetin isolated from *C. mimosoides* showed anticholinesterase and neuroprotective properties (Tangsaengvit et al., 2013).

In our previous work we have evaluated antimicrobial, antioxidant and wound healing properties of *C. mimosoides* crude ethanol extract (Bhat et al., 2016). In continuation, the present study was taken up to enrich the ethanol extract for phenolic compounds through sequential liquid-liquid fractionation procedure. The phenol enriched fraction (PEF) was analyzed for *in vitro* antimicrobial activity with bacterial skin pathogens and fungal dermatophytes, followed by *in vitro* antioxidant and anti-inflammatory activities. The PEF was further evaluated for its safety parameters through *in vitro* viability and toxicity assays against L929, 3T3L1 and L6 cell lines and *in vivo* acute toxicity studies in Wistar rat model. Wound healing activity was carried out through *in vitro* wound scratch assay using the cell lines previously mentioned and *in vivo* circular excision and paravertebral incision wound models in Wistar rats, followed by biochemical parameters of granulation tissues. Subsequently, identification of phytoconstituents was performed using GC-MS and RP-UFLC-DAD instrumentation methods.

## 2 Materials and methods

### 2.1 Collection of plant material, extraction and fractionation for the enrichment of phenols

Fresh tender shoots of *C. mimosoides* were collected from Yana village located in Central Western Ghats region, Uttara Kannada district of Karnataka state, India (14° 34′ 0″ N and 74° 32′ 59″ E). The plant was authenticated with standard floras and deposited the voucher specimen (PB/GRH-111) in the Herbarium, Karnatak University, Dharwad for further references.

The collected plant material (2.5 kg fresh weight) was cleaned; shade dried and coarse powdered in an electric grinder. The powdered plant material (1 kg) was sequentially extracted in n-hexane, chloroform, acetone, ethanol and water through hot percolation method using Soxhlet apparatus as described in our previous work (Bhat et al., 2016). Among all, the active ethanol extract was further fractionated for enrichment of phenols as described by Das et al. (2012). In brief, the dried ethanol extract (40 g) was suspended in water and subjected to sequential liquid-liquid extraction with equal volume of n-hexane (4 × 200 ml), chloroform (3 × 200 ml) and ethyl acetate (5 × 200 ml) using separating funnel. The solution was allowed to stand for 2 h for complete liquid-liquid separation. Each step was repeated thrice to ensure the complete extraction in each case. The fractions (n-hexane, chloroform and ethyl acetate) were col l ected separately and subjected to solvent evaporation using rotary evaporator (Heidolph Laborota 4000), weighed and stored in glass vials, maintained at 4°C in the refrigerator for further use.

The percentage yield of each fraction was calculated with the formula mentioned below:

$$\%\;Yield=\frac{Weight\;of\;the\;enriched\;fraction\;obtained}{Weight\;of\;crude\;ethanol\;extract\;taken}\times 100$$

### 2.2 Estimation of total phenol and flavonoid contents in fractions

Total phenol content of all three fractions was determined through Folin-Ciocalteu reagent method with minor modifications described by Ghane et al. (2018). A volume of 200 μl fractions (mg/ml) was added to 1 ml Folin-Ciocalteu reagent and properly mixed. The reaction mixture was incubated for 5 min, 0.8 ml (7.5% w/v) of sodium carbonate was added and allowed to stand for 60 min at room temperature. The absorbance was measured at 765 nm using 96 well microplate reader (Thermo Scientific Multiscan Go Version 1.00.40). Different concentrations of gallic acid solution (1,000–3.9 μg/ml) was used to plot the standard calibration curve and the total phenolic content in each fraction was expressed as mg gallic acid equivalent (GAE) per gram fraction (d.w.).

Total flavonoid content of fractions was estimated by aluminium chloride colorimetric method (Tohidi et al., 2017). A volume of 250 μl fractions (mg/ml) was added to 150 μl of 5% sodium nitrite solution and allowed to stand for 5 min, followed by the addition of 300 μl aluminium chloride (10%). A volume of 1.5 ml sodium hydroxide solution (1 M) was added to the reaction mixture after 5 min of incubation and the final volume was made up to 5 ml with methanol. The absorbance of the reaction mixture was measured after 15 min of incubation at 510 nm using 96 well microplate reader. A standard quercetin solution (1,000–15.62 μg/ml) was used for plotting the calibration curve and the total flavonoid content in each fraction was expressed as mg quercetin equivalent (QE) per gram fraction (d.w.).

The fraction with higher phenol and flavonoid contents was considered as phenol enriched fraction (PEF) and it was taken up for further studies.

### 2.3 Antioxidant activity *in vitro*

#### 2.3.1 DPPH radical scavenging assay

DPPH activity of PEF was calculated following Brand-Williams et al. (1995). DPPH of 0.1 mM solution (3.5 ml) prepared in methanol was added to 0.5 ml varying concentrations of standard quercetin and PEF dissolved in methanol (100–1.56 μg/ml). After incubating the reaction mixture for 30 min at 37°C, the absorbance was measured at 517 nm against control (DPPH solution) in 96 well microplate reader.

#### 2.3.2 Nitric oxide radical scavenging activity

Griess Illosvoy reaction procedure (Hazra et al., 2008) was followed to measure the Nitric oxide radical scavenging activity of PEF. Sodium nitroprusside (2 ml; 10 mM) in 0.5 ml phosphate buffer (p^H^ 7.4; 0.5 M) was mixed with 0.5 ml varying concentrations (1,000–15.6 μg/ml) of standard ascorbic acid and PEF. The reaction mixture was further incubated for 150 min at 25°C. Sulfanilamide (0.33% in 20% glacial acetic acid; 1 ml) was added to 0.5 ml reaction mixture and incubated for 5 min. Pink chromophore was generated by adding naphthylethylenediamine dihydrochloride (1.0 m; 0.1% w/v). The reaction mixture was incubated for 30 min and the absorbance was measured against control at 540 nm, in 96 well microplate reader.

IC_50_ of PEF against DPPH and nitric oxide radical scavenging activities were also determined through standard calibration curve equation using ED50 plus v1.0 software program.

### 2.4 Anti-inflammatory activity of PEF *in vitro*

#### 2.4.1 Protein denaturation inhibition assay

The assay was carried out following the method described by Anwar et al. (2020) with minor modifications. Bovine serum albumin (1% aqueous solution; 450 µl) was added to 50 µl of standard diclofenac sodium and PEF with varying concentrations (500–50 μg/ml). Initially the reaction mixture was incubated for 20 min at 37°C and further heated (51°C) up to 20 min. The turbid reaction mixture was cooled and absorbance was measured against reagent blank (distilled water) at 660 nm using 96 well microplate reader.

#### 2.4.2 Proteinase inhibitory activity

The proteinase inhibitory activity of PEF was determined by adding 0.06 g trypsin, 1 ml tris-HCl buffer (20 mM; pH 7.4) in 0.5 ml phosphate buffer (0.5M; p^H^ 7.4) with 2 ml varying concentrations of standard diclofenac sodium and PEF (500–50 μg/ml each, dissolved in methanol) (Oyedepo and Femurewa, 1995). The reaction mixture was incubated for 5 min (37°C) and added 1 ml casein (0.8% w/v), sodium carbonate solution (5 ml; 500 mM) and 1 ml Folin-Ciocalteu’sreagent (10%). Incubated the reaction mixture for further 20 min and the reaction was arrested by adding 70% perchloric acid (2 ml). Centrifuged the turbid suspension at 2,500 rpm for 5 min and absorbance of the supernatant was recorded at 210 nm using tris-HCl buffer as blank using 96 well microplate reader.

Protein denaturation inhibition and proteinase inhibitory activity (% inhibition) of PEF and standard diclofenac sodium were calculated using the following equation:

$$\%\;inhibition=\left[\frac{Absorbance\;of\;control-Absorbance\;of\;sample}{Absorbance\;of\;control}\right]\times 100$$

Further, IC_50_ of PEF and the standard diclofenac sodium were also determined through standard calibration curve equation using ED50 plus v1.0 software program.

### 2.5 Antimicrobial activity of PEF *in vitro*

Antimicrobial activity of PEF was performed by minimum inhibitory concentration assay through tube dilution method (Bhat et al., 2016) and disc diffusion assay (Perianayagam et al., 2012) against seven bacterial skin pathogens and seven fungal dermatophytes. The standard strains were obtained from Microbial Type Culture and Collection (MTCC), Chandigarh, India and National Collection of Industrial Microorganisms (NCIM), Pune, India. The bacterial strains such as *Staphylococcus aureus* (MTCC737), *Proteus* vulgaris (MTCC 1771), *Klebsiella pneumoniae* (MTCC 109), *Pseudomonas aeruginosa* (MTCC 1688), *Micrococcus* flavus (NCIM 2379), *Salmonella typhimurium* (NCIM 2501) and *Micrococcus* luteus (NCIM 2103), followed by the fungal strains Trichophyton mentagrophytes (MTCC 7687), Trichophyton rubrum (MTCC 296), *Candida* albicans (MTCC 183), *Microsporum canis* (MTCC 2820), *Microsporum gypseum* (MTCC 2819), *Malassezia furfur* (MTCC 1374) and *Epidermophyton floccosum* (MTCC 7880) were used to assess the antimicrobial activity of PEF. Nutrient Agar, Sabouraud Dextrose Agar and Potato Dextrose Agar media were used to subculture the bacterial and fungal strains.

#### 2.5.1 Determination of antimicrobial activity through tube dilution method

The bacterial and fungal suspension cultures were prepared in peptone water and incubated at 37°C for 18–24 h and the turbidity of suspension cultures were adjusted to 0.5 McFarland standard units (1.5 × 10^6^ CFU/ml). The activity was compared against reference standard Streptomycin (Himedia Laboratories Limited, Mumbai) for bacterial strains; Nystatin and Voriconazole (Himedia Laboratories Limited, Mumbai) for fungal strains. The stock solutions of PEF and the reference standards were in mg/ml, dissolved in 1% DMSO and serial two-fold dilutions were made ranging between 1,000 and 0.97 μg/ml. Incubated the tubes for 24–48 h at 37°C and the lowest concentration of PEF that inhibited the growth of microbial strains was treated as MIC (Bhat et al., 2016).

#### 2.5.2 Determination of antimicrobial activity through disc diffusion method

The bacterial and fungal cell suspension cultures were prepared in peptone water and incubated for at 37°C for 18–24 h and the turbidity of suspension cultures were adjusted to 0.5 McFarland standard units (1.5 × 10^6^ CFU/ml) before inoculating into the petri plates of respective media. Antimicrobial activity was performed using 10 µl of different concentrations of PEF (10, 25 and 50 mg/ml) on sterile disc (Himedia Laboratories Limited, Mumbai) and 1% DMSO was used as negative control. Streptomycin (10 µg/disc) was used as a reference standard for bacterial strains and Nystatin (100 units/disc), Voriconazole (1 µg/disc) and Fluconazole (25 µg/disc) for fungal strains. Petriplates were incubated for a period of 24 h at 37°C for bacteria and 48 h at 30°C for fungi. Antimicrobial activity was measured as diameter (mm) of the clear zone of inhibition (ZI) formed around the disc with Hi Antibiotic Zone Scale™ (Himedia Laboratories Limited, Mumbai) (Perianayagam et al., 2012).

### 2.6 *In vitro* cell viability/cell toxicity assay

L929 mouse fibroblast cells, 3T3L1 mouse adipocyte embryo cells and L6 rat pre- myoblast cells were obtained from National Centre for Cell Science (NCCS), Pune, India. All the cells were maintained at the conditions as per manufacturer’s instructions. L929 cells were maintained in Dulbecco’s Modified Eagle’s Medium (DMEM), 10% fetal bovine serum (FBS) and 1% penicillin-streptomycin. 3T3L1 and L6 cells were maintained in DMEM with 4 mM l-glutamine, 1.5 g/L sodium bicarbonate and 4.5 g/L glucose, FBS 10%. Cells were maintained at 37°C, 5% CO_2_ in humidified incubator. Cell viability was determined by MTT assay (3-[4,5-Dimethylthiazol-2-yl]-2,5- diphenyltetrazolium bromide) (Azis et al., 2017). The cells were seeded at 1×10^4^ cell density in a 96-well microplate (Nunc^TM^Microwell^TM^96-well microplates, ThermoFisher Scientific) containing respective media in each well, followed by overnight incubation. The growth media was replaced with fresh serum-free media with different concentrations of PEF (1,000–1.95 μg/ml) and the cells treated with serum free media without PEF was considered as control cells. After 24 h of incubation period, a serum-free medium containing 25 µl of 5 mg/ml MTT was added to the cells and incubated for 4 h at 37°C. The medium was discarded, and the wells were completely dried. Dimethyl sulfoxide solution (100 μl) was added to each well to dissolve residual formazan crystals and the absorbance was measured spectrophotometrically at 540 nm by using 96 well microplate reader. The percentage cell viability and toxicity were calculated by using following equation:

$$\%\;viability=\left[\frac{Absorbance\;of\;sample}{Absorbance\;of\;control}\right]\times 100$$

$$\%\;toxicity=\left[\frac{Absorbance\;of\;control-Absorbance\;of\;sample}{Absorbance\;of\;control}\right]\times 100$$

Further, IC_50_ of PEF was also determined through standard calibration curve equation using ED50 plus v1.0 software program.

### 2.7 *In vitro* wound healing activity (wound scratch assay)

L929, 3T3L1 and L6 cells were seeded into 24-well plates containing respective medium at 37°C and 5% CO_2_ (Azis et al., 2017). A linear wound was created using a 10 μl sterile pipette tip on the confluent monolayer of cells and phosphate buffer saline (PBS) was used to wash the wells to remove cell debris. The wells with plane medium (negative control) and three different concentrations of PEF which showed more than 100% viability in respective cell lines were considered as the treatment groups. Epidermal growth factor (EGF) at 0.002 μg/ml concentration was used as positive control. Images of the scratch were taken at 0 h, 12 h, 24 h and 48 h using a phase contrast inverted microscope (Olympus CKX41) attached to Magcam DC5 camera with MagVision software. The images at 10× magnifications were photographed to estimate the relative cell migration of cells. The area enclosed between the scratch edges was measured using NIH ImageJ software. The percentage wound contraction was measured using following equation:

$$\%\;wound\;contraction=\left[\frac{Wound\;area\;at{\;}^{\prime }0^{\prime }th\;hour-Wound\;area\;at{\;}^{\prime }n^{\prime }th\;hour}{Wound\;area\;at\;0th\;hour}\right]\times 100$$

### 2.8 *In vivo* acute dermal toxicity studies

The dermal toxicity study provides information on possible health hazards likely to be arising from short-term and repeated exposures of test substances by the dermal route over a limited period of time. Protocols of the experiments were approved by the Ethical Committee, Soniya College of Pharmacy, Dharwad (SETCP/IAEC/2012-13/519). Wistar rats of both sexes (200–280 g body weight) were selected for assessing acute toxicity studies to determine the safe dose in compliance with OECD guidelines (OECD, 2017). The animals were maintained under standard conditions, supplied with Amrut Laboratory animal feeds, Pune, India and water *ad libitum* for the entire experimental period. The PEF was dissolved in simple ointment base (as per Indian Pharmacopoea) to prepare the test doses, where it was used as vehicle control. After 7 days of acclimatization period, fur from the dorsal trunk of the test animals was removed using electric clipper, approximately 24 h before the test. A limit test at one dose level of 2000 mg/kg b.w. was carried out in 20 rats randomly divided into two groups (control and test group), each group with five male and five female rats respectively. The vehicle control group (Group I) and the test group (Group II) was topically applied with ointment base and PEF ointment once at 2000 mg/kg b.w. The animals were continuously observed upto 14 days for any toxic symptoms on rat skin, such as irritation, itching, redness, swelling and other behavioral patterns.

### 2.9 *In vivo* wound healing activity

The healthy adult Wistar albino rats (200–280 g) of either sex were used for the experiment. Protocols of the experiments were approved by the Ethical Committee, Soniya College of Pharmacy, Dharwad (SETCP/IAEC/2012-13/519). The animals were maintained under standard conditions: temperature (25 ± 2°C), relative humidity (55%) and alternating light-dark cycle (12 h/12 h), supplied with Amrut Laboratory animal feeds, Pune, India and water *ad libitum* for the entire experimental period. Animals were placed individually in separate cages of corresponding groups after 7 days of acclimatization. Circular excision and para-vertebral linear incision wound models were used to evaluate the wound healing activity of PEF as per the standard method (Bhat et al., 2016). Different concentrations of PEF samples (2.5% and 5%) were prepared by using simple ointment base formulation (wool fat, hard paraffin, cetostearyl alcohol and yellow soft paraffin in 1:1:1:7 ratio) as per Indian Pharmacopoeia (IP) (Biswas et al., 2013). The sample size (n) was calculated statistically by power analysis method using G* Power statistical software program (Charan and Kantharia, 2013) and was finalized as *n* = 6 per group.

The animal groupings for both circular excision and para-vertebral linear incision wound models were as below:

Group I- Test ointment group (Ointment containing PEF 2.5% w/w)

Group II- Test ointment group (Ointment containing PEF 5% w/w)

Group III- Positive control group (Cipladine ^®^ Povidone-Iodine ointment USP 5% w/w)

Group IV- Ointment base group [Ointment base (IP)]

Group V- Negative control group (Without any treatment)

#### 2.9.1 Circular excision wound model

Animals were anaesthetized prior to wound creation by following open mask procedure (Bhat et al., 2016). The fur on dorsal surface of each animal was shaved 1 day prior to the experiment with electric clipper. A circular full thickness excision wound (approximately 500 mm^2^ diameter and 2 mm depth) was made on the shaved back of the rats with sterilized stainless steel surgical scissor. The wound was left open and topically applied with standard Povidone-Iodine and test ointments (dose-1g/wound) once a day to the respective groups of animals till 15th post-wounding day. The day of wound creation was considered as day “0” and the raw wound was traced with sterilized transparent polythene paper and permanent marker on every alternate day up to 15th post wounding days. The traced polythene paper was transferred to a millimeter scale graph paper and wound area was measured. Photographs of the wounds were taken using high resolution digital camera (Sony Cyber-shot). Further, wound contraction percentage (Arunachalam ans Parimelazhagan, 2013) was calculated using following formula:

$$Wound\;contraction\;\%=\left[\frac{Wound\;area\;on\;day{\;}^{\prime }0^{\prime }–\;Wound\;area\;on\;day{\;}^{\prime }n^{\prime }}{Wound\;area\;on\;day{\;}^{\prime }0^{\prime }}\right]\times 100$$

where, *n* = wound area on first, third, fifth, seventh, ninth, 11th, 13th and 15th post wounding days

The Complete epithelization period (CEP) of all the treatment groups was also determined following the method of Fikru et al. (Fikru et al., 2012).

#### 2.9.2 Linear incision wound model

A 6 cm length and 2 mm depth longitudinal para-vertebral incision wound was created by following open mask procedure (Bhat et al., 2016) on the shaved back of the rats. The parted skin was sutured with stainless steel disposable skin staplers (Acos™ Sunmedix) at 1 cm intervals. The wounds were topically applied with standard Povidone-Iodine and test ointments (dose-1g/wound/day) for the period of 15 days. The skin staplers were removed on 13th post-wounding day with continued ointment application. Animals were sacrificed under anesthesia on 15th day and the tensile strength (weight in grams required to break open the mm^2^ area of skin wound) of the animal skin was measured through Tensiometer instrument (Lee., 1968). The amount of weight (in grams) required to break open the mm^2^ area of incised wound was considered as tensile strength (g/mm^2^).

#### 2.9.3 Histopathological examinations of granulation tissues

Skin samples of the rats from each treatment group of excision wound model were collected on 15th post-wounding day for further histopathological studies. Sections of 5 µm thickness of fixed and processed skin samples were stained with hematoxylin & eosin (H&E), Toluidine Blue and Masson’s trichome stains (Suntar et al., 2010). The sections were analyzed through microphotographs using a compound microscope (Carl Zeiss Axio Imager M2) at different magnifications (×100 and ×400) with the inbuilt analog camera (ProgRess^®^ C5-JENOPTIK). H&E stained sections were examined to evaluate the epidermal or dermal re-modeling such as re-epithelization or ulcus in epidermis, fibroblast proliferation, formation of blood capillaries, neovascularisation, collagen deposition and other angiogenesis process. Masson’s trichome staining was performed for differential staining of collagen deposition and other connective tissues with keratin, muscle fibers, cytoplasm and cell nuclei. Whereas sections stained with Toluidine blue were analyzed for metachromatic staining of mast cells. The histological parameters were graded as nil (-), mild (+), moderate (++) and severe (+++) for epidermal or dermal re-modeling (Suntar et al., 2011). Cell population at the wound site i.e. inflammatory cells, myofibroblasts, macrophages, blood vessels, neutrophils and mast cells in the granulation tissue sections were quantified using NIH ImageJ software (Hashemnia et al., 2019).

#### 2.9.4 Estimation of connective tissue, enzymatic antioxidants, non-enzymatic antioxidants and oxidative stress parameters in the granulation tissues

Biochemical studies of the granulation tissues were performed to analyze connective tissue parameters such as hydroxyproline (Bergman and Loxley, 1963) and hexosamine (Cheng, 1963); enzymatic antioxidants such as superoxide dismutase, SOD (Kakkar et al., 1984) and catalase, CAT (Hadwan and Ali, 2018); non-enzymatic antioxidants *viz.* reduced glutathione, GSH (Moron et al., 1979) and oxidative stress parameter lipid peroxidation, LPO (Astaneie et al., 2005). Each granulation tissue sample was dried and weighed prior to carry out the analysis. Tissues were homogenized using a manual homogenizer with suitable solvents as per the standard protocols. The homogenized tissues were then centrifuged at 6,000 rpm for 20 min and supernatants were carried out for further biochemical estimations.

### 2.10 GC-MS analysis

GC-MS analysis of PEF was carried out using Gas Chromatography (Perkin Elmer Clarus 680 and Mass spectrometry (Perkin Elmer Clarus 600 EI), equipped with a Rtx-5MS, fused silica capillary column of 5% diphenyl and 95% dimethylpolysiloxane (30 m × 0.25 mm) ID × 1 µm. Helium was used as carrier gas at a constant flow of 1.20 ml/min. The injector temperature, ion source temperature and interface temperature were set to 260°C, 220°C and 280°C respectively, with splitless injection mode. Oven temperature program was initially set to 60°C (hold time 2 min), followed by 240°C (10th min; hold time 2 min) and 280°C (15th min; hold time 20 min). Ionization mode was at 70 eV, with a scan time 0.20 s with a mass range of 40–650 Da. The total GC run time was 30 min.

Identification of the components was achieved on the basis of Retention Index (RI, determined with reference to homologous series of n-alkanes C8–C25, under identical experimental conditions), MS library search (NIST 08 Mass Spectra Library- Version 2.0f, WILEY’s Library of Mass Spectra ninth Edition), and by comparison with MS literature data (Adams, 2007). The relative amounts of individual components were calculated based on the GC peak area without using correction factor.

### 2.11 RP-UFLC-DAD analysis

Reverse phase-ultra flow liquid chromatography-diode array detector (RP-UFLC-DAD) analysis of PEF was achieved in Shimadzu system (Model number: LC-20AD), supplied with manual injector, four channelled pump, DGU-20A5 degasser and diode array detector (Model No. SPD-M20A). The built-in Liquid chromatography (LC) solution software system was used for data processing and Qualisil BDS C18 column (250 × 4.6 mm; 5 μm) was used for chromatographic separation. Acetonitrile, water and glacial acetic acid (12:85:3 volume: volume: volume; pH—2.6) solvent was used as mobile phase in an isocratic mode with an injection volume of 20 μl. The flow rate was set at 1 ml/min with 25 min run time for both standard and samples. The wavelength of Diode array detector (DAD) was set at 271 nm and known quantities of standards and samples were dissolved in methanol (mg/ml stock solution) and serially diluted to get eight different concentrations of standards (0.5–100 μg/ml) to draw the standard calibration curve. Three replicates of standard compounds at different concentrations were run to assess the system suitability test. As per ICH guidelines, the limit of detection (LOD) and limit of quantification (LOQ) were analyzed and they were found with 3.3 and 10 signal/noise ratio respectively (ICH guidelines, 2005). The concentration of the compounds in the samples was calculated through standard calibration curve equation and was expressed as μg/mg of extract dry weight (dw).

### 2.12 Statistical analysis of the data

All the experimental parameters were conducted in triplicates and the values were expressed as Mean ± Standard error. Results were analyzed with one way ANOVA using IBM SPSS software package of 20th version. Statistical significance of differences of the experimental results was analyzed by Tukey post hoc test at *p* ≤ 0.05, *p* ≤ 0.01 and *p* ≤ 0.001.

## 3 Results

### 3.1 Percent yield of fractions

The yield of completely dried fractions was in the range of 1.32%–28.90%. Highest yield was in ethyl acetate fraction (28.90 ± 0.64%), followed by chloroform (4.97 ± 0.19%) and n-hexane (1.70 ± 0.24%) respectively.

### 3.2 Total phenol and flavonoid contents

Total phenol contents in all the fractions were determined through the calibration curve equation Y = 0.0044x+0.0569, *R* ^2^ = 0.99. Among all, ethyl acetate fraction showed significantly higher phenolic content [578.28 ± 2.30 mg GAE/g of fraction (d.w.)], whereas it was 33.43 ± 1.64 and 28.43 ± 1.51 mg GAE/g of fraction (d.w.) for chloroform and n-hexane fractions respectively. Similarly, the total flavonoid contents of fractions were calculated according to the calibration curve equation Y = 0.0007x-0.0062, *R* ^2^ = 0.99. Among all, the ethyl acetate fraction exhibited significantly higher quantity of total flavonoid content [270.76 ± 2.52 mg QE/g of fraction (d.w.)] compared to other two fractions (Table 1).

**TABLE 1:** Total phenol and flavonoid contents in n-hexane, chloroform and ethyl acetate fractions.

Fractions	Total polyphenol content (mg GAE/g extract d.w.) (Mean ± S.E.)	Total flavonoid content (mg QE/g extract d.w.) (Mean ± S.E.)
n-Hexane	28.43 ± 1.51^a^	10.76 ± 0.47^a^
Chloroform	33.43 ± 1.64^a^	15.05 ± 0.95^a^
Ethyl acetate	578.28 ± 2.30^b^	270.76 ± 2.52^b^

S.E, Standard error. Different letters (^a,b^) in each column represent significant differences between the variables of associated groups through Tukey’s *Post hoc* analysis at *p* ≤ 0.05.

Since ethyl acetate fraction showed significantly higher quantities of total phenol and flavonoid contents, it was considered as phenol enriched fraction (PEF) and taken up for further activities.

### 3.3 *In vitro* antioxidant activity

DPPH activity of standard quercetin and PEF showed dose-dependent increase in the percentage scavenging from 26.25 ± 0.01% to 90.60 ± 0.04% and 25.15 ± 0.01% to 88.57 ± 0.06% respectively at 1.56 and 100 μg/g concentrations. Both standard quercetin and PEF showed almost equivalent IC_50_ results with 12.40 ± 0.19 and 16.39 ± 0.18 μg/g respectively (Supplementary Figures S1A, S1C).

Similarly, nitric oxide radicals were scavenged by increased concentration of standard ascorbic acid and PEF. Ascorbic acid exhibited 96.22 ± 0.12% inhibition at 1,000 μg/g concentration and PEF was also active with 93.39 ± 0.21% inhibition at the same concentration. Moreover, IC_50_ of both standard ascorbic acid and PEF were also close to each other (111.67 ± 3.11 μg/g and 124.41 ± 2.06 μg/g respectively) (Supplementary Figures S1B, S1C).

### 3.4 *In vitro* anti-inflammatory activity of PEF

PEF exhibited significant anti-inflammatory activity with dose dependent increase in the percentage inhibition of both protein denaturation and proteinase inhibition. It was in the range of 14.01 ± 0.44%-80.50 ± 0.56% and 31.64 ± 0.68%-84.84 ± 0.21% at 50–500 μg/g concentrations respectively. However, the standard diclofenac sodium exhibited 24.84 ± 0.77%-88.43 ± 0.16% and 37.21 ± 0.25%-89.72 ± 0.52% at 50–500 μg/g concentrations respectively (Supplementary Figures S2A, S2B). The corresponding IC_50_ of PEF was 202.22 ± 1.09 and 78.23 ± 4.70 μg/g, which was moderately comparable to the standard diclofenac sodium (175.98 ± 4.95 and 69.28 ± 4.78 μg/g) (Supplementary Figure S2C).

### 3.5 Antimicrobial activity of PEF *in vitro*

PEF showed broad spectrum of antimicrobial activity against most of the bacterial and fungal strains tested. Amongst all the bacterial strians, PEF was significantly effective against M. luteus with least MIC and highest ZI (MIC: 7.81 ± 0.00 μg/ml; ZI: 35.33 ± 0.67 mm), compared to the standard streptomycin (MIC: 2.60 ± 1.12 μg/ml; ZI: 25.67 ± 0.33 mm). It was followed by M. flavus (MIC: 41.67 ± 10.42 μg/ml; ZI: 12.67 ± 0.33 mm), P. vulgaris (MIC: 41.67 ± 10.42 μg/ml; ZI: 18.33 ± 0.67 mm), *P. aeruginosa* (MIC: 52.08 ± 10.42 μg/ml; ZI: 19.67 ± 0.33 mm), *S. aureus* (MIC: 52.08 ± 10.42 μg/ml; ZI: 13.67 ± 0.33 mm) and *S. typhimurium* (MIC: 125.00 ± 0.00 μg/ml; ZI: 6.67 ± 0.33 mm) (Table 2).

**TABLE 2:** Antibacterial activity of PEF through tube dilution method (MIC) and disc diffusion method (ZI).

PEF/Positive control	Tube dilution method MIC (µg/ml) Mean ± S.E.						
	*P. aeruginosa*	*S. typhimurium*	*P. vulgaris*	*S. aureus*	*K. pneumoniae*	*M. luteus*	*M. flavus*
PEF	52.08 ± 10.42^a^	125.00 ± 0.00^a^	41.67 ± 10.42^a^	52.08 ± 10.42^a^	125.00 ± 0.00^a^	7.81 ± 0.00^a^	41.67 ± 10.42^a^
Streptomycin	6.50 ± 1.30^b^	5.20 ± 1.30^b^	3.90 ± 0.00^b^	10.41 ± 2.60^b^	5.20 ± 1.30^b^	2.60 ± 1.12^b^	2.60 ± 1.12^b^
**PEF/Positive control**	**Disc diffusion method**
	**Zone of inhibition (mm); Mean ± S.E.**						
	***P. aeruginosa***	***S. typhimurium***	***P. vulgaris***	***S. aureus***	***K. pneumoniae***	***M. luteus***	***M. flavus***
10 mg/ml	13.33 ± 0.67^a^	6.00 ± 0.00^a^	11.33 ± 0.67^a^	9.67 ± 0.33^a^	6.33 ± 0.33^a^	21.33 ± 0.67^a^	8.67 ± 0.67^a^
25 mg/ml	17.33 ± 0.67^b^	6.33 ± 0.33^a^	15.33 ± 0.67^b^	13.67 ± 0.33^b^	6.67 ± 0.33^a^	28.67 ± 0.67^b^	10.67 ± 0.33^b^
50 mg/ml	19.67 ± 0.33^b^	6.67 ± 0.33^a^	18.33 ± 0.67^c^	13.67 ± 0.33^b^	7.67 ± 0.33^a^	35.33 ± 0.67^c^	12.67 ± 0.33^c^
Streptomycin (10 µg/disc)	26.67 ± 0.33^c^	19.67 ± 0.33^b^	22.33 ± 0.67^d^	18.67 ± 0.33^c^	24.67 ± 0.33^b^	25.67 ± 0.33^d^	23.00 ± 0.00^d^

S.E., Standard error. Different letters (^a,b,c,d^) in each column represent significant differences between the variables of associated groups by Tukey Post hoc analysis at *p* ≤ 0.05.

Similarly, PEF was found effective against the fungal skin pathogen M. gypseum with least MIC and highest ZI (MIC: 15.62 ± 0.00 μg/ml; ZI: 24.67 ± 0.33 mm), compared to the standard voriconazole (MIC: 1.69 ± 0.97 μg/ml; ZI: 25.33 ± 0.33 mm). The other strains which were moderately susceptible to PEF are T. rubrum (MIC: 20.83 ± 5.21 μg/ml; ZI: 22.33 ± 0.33 mm), followed by C. albicans (MIC: 31.25 ± 0.00 μg/ml; ZI: 20.33 ± 0.67 mm), T. mentagrophytes (MIC: 31.25 ± 0.00 μg/ml; ZI: 20.67 ± 0.33 mm), M. furfur (MIC: 83.33 ± 20.83 μg/ml; ZI: 17.33 ± 0.67 mm) and *M. canis* (MIC: 104.17 ± 20.83 μg/ml; ZI: 10.67 ± 0.33 mm); whereas, E. floccosum was found resistant against PEF without any inhibitory activity (MIC more than 250 μg/ml) (Table 3).

**TABLE 3:** Antifungal activity of PEF through tube dilution method (MIC) and disc diffusion method (ZI).

PEF/Positive controls	Tube dilution method MIC (µg/ml) Mean ± S.E.						
	*T. rubrum*	*T. mentagrophytes*	*M. canis*	*M. gypseum*	*E. floccosum*	*M. furfur*	*C. albicans*
PEF	20.83 ± 5.21^a^	31.25 ± 0.00^a^	104.17 ± 20.83^a^	15.62 ± 0.00^a^	≥250	83.33 ± 20.83^a^	31.25 ± 0.00^a^
Nystatin	3.25 ± 0.65^b^	6.51 ± 1.30^b^	5.20 ± 1.30^b^	2.60 ± 0.65^b^	5.20 ± 1.30^b^	5.20 ± 1.30^b^	2.60 ± 0.65^b^
Voriconazole	1.62 ± 0.32^b^	1.95 ± 0.97^c^	1.95 ± 0.97^b^	1.69 ± 0.97^b^	3.25 ± 0.65^b^	3.25 ± 0.65^b^	4.55 ± 1.72^b^
**PEF/Positive control**	**Disc diffusion method**
	**Zone of inhibition (mm); Mean ± S.E.**						
	***T. rubrum***	***T. mentagrophytes***	***M. canis***	***M. gypseum***	***E. floccosum***	***M. furfur***	***C. albicans***
10	17.33 ± 0.67^a^	14.33 ± 0.67^a^	8.33 ± 0.67^a^	17.33 ± 0.67^a^	NA	12.33 ± 0.33^a^	14.67 ± 0.33^a^
25	19.67 ± 0.33^b^	18.67 ± 0.33^b^	8.67 ± 0.33^a,b^	21.33 ± 0.67^b^	NA	15.33 ± 0.67^b^	18.33 ± 0.67^b^
50	22.33 ± 0.33^c^	20.67 ± 0.33^c^	10.67 ± 0.33^b^	24.67 ± 0.33^c^	NA	17.33 ± 0.67^b^	20.33 ± 0.67^b^
Nystatin (100units/disc)	18.67 ± 0.33^a,b^	23.67 ± 0.33^d^	13.33 ± 0.33^a^	16.00 ± 0.58^a,d^	16.67 ± 0.88^a^	20.67 ± 0.33^c^	29.67 ± 0.33^c^
Voriconazole (1 μg/ml)	27.33 ± 0.33^d^	34.67 ± 0.33^e^	23.00 ± 0.58^b^	25.33 ± 0.33^c,e^	33.67 ± 0.88^b^	30.00 ± 0.58^d^	32.33 ± 0.67^d^

S.E, standard error, NA, No activity. Different letters (^a,b^) in each column represent significant differences between the variables of associated groups by Tukey Post hoc analysis at *p* ≤ 0.05.

### 3.6 *In vitro* cell viability/cell toxicity assay

The viability of L929, 3T3L1 and L6 cells treated with different concentrations of PEF through MTT assay is presented in Supplementary Figure S3. It was found that increasing concentration of PEF led to gradual decrease in viability of all the cell lines. The values of percentage cell viability ranged between 34.73 ± 0.43 to 100.18 ± 1.37% (L929), 16.37 ± 0.45 to 114.76 ± 1.65% (3T3L1) and 1.22 ± 0.32 to 102.47 ± 2.14% (L6) at the concentrations of 1,000 μg/ml to 1.95 μg/ml respectively (Supplementry Figure S3A). Dose-dependent cell viability indicated that more than 90% of cell viability was observed at ≤7.81 μg/ml concentration for L6 and ≤15.62 μg/ml for both L929 and 3T3L1 cells respectively. The IC_50_ of cell toxicity was found to be 350.83 ± 1.35, 323.52 ± 2.03 and 251.29 ± 12.23 μg/ml for L929, 3T3L1 and L6 cells respectively (Supplementary Figure S3B).

### 3.7 *In vitro* wound healing activity (wound scratch assay)

**FIGURE 1:**
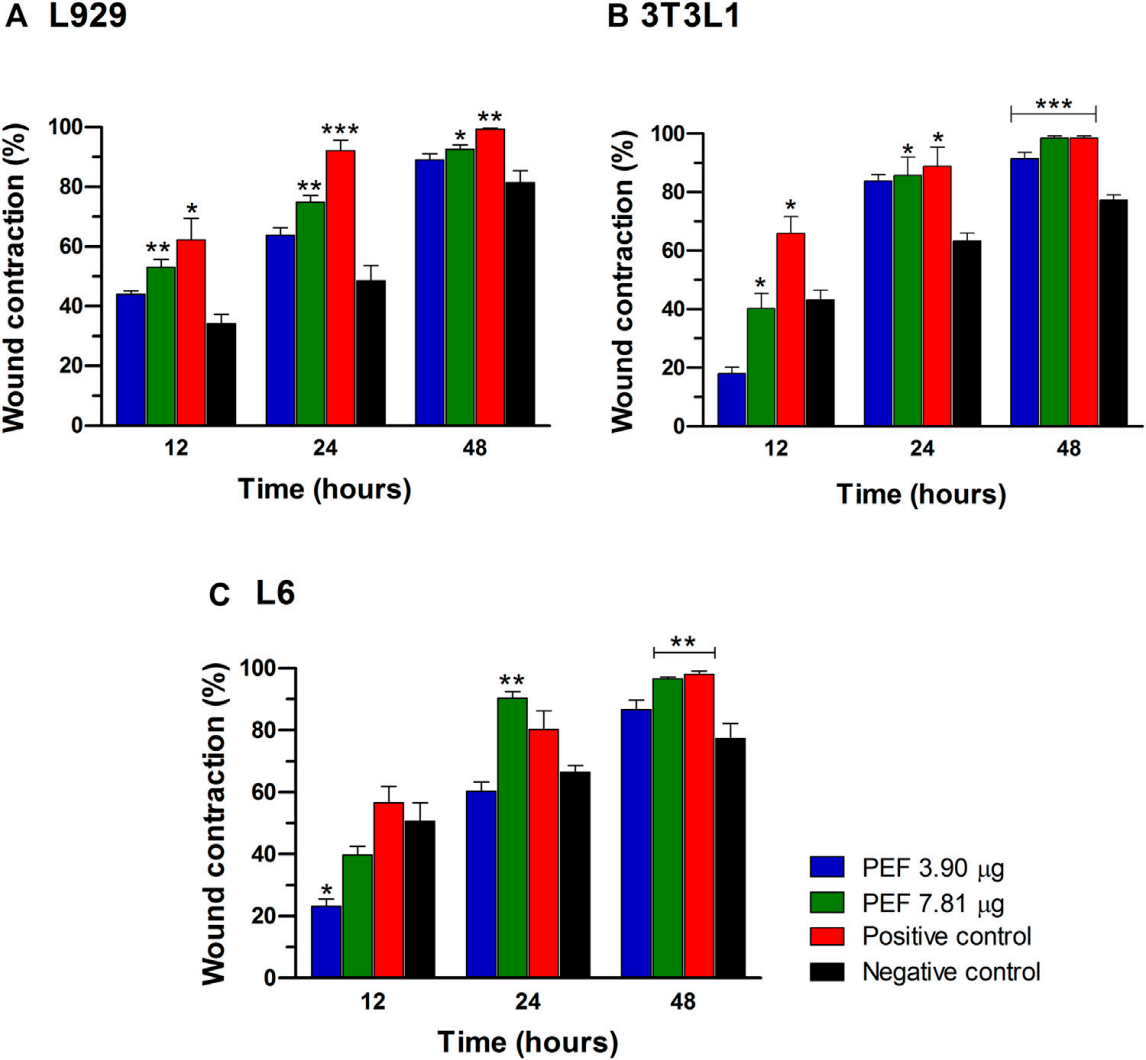
Percentage cell migration/wound contraction in wound scratch assay. **(A)** L929 cells, **(B)** 3T3L1 cell line, **(C)** L6 cells. Significant differences at ^*^ *p* ≤ 0.05, ^**^ *p* ≤ 0.01 and ^***^ *p* ≤ 0.001 between negative control and other treatment groups were noted at different post wounding time (hours). The data was analyzed using one-way ANOVA with Tukey *post hoc* test.

**FIGURE 2:**
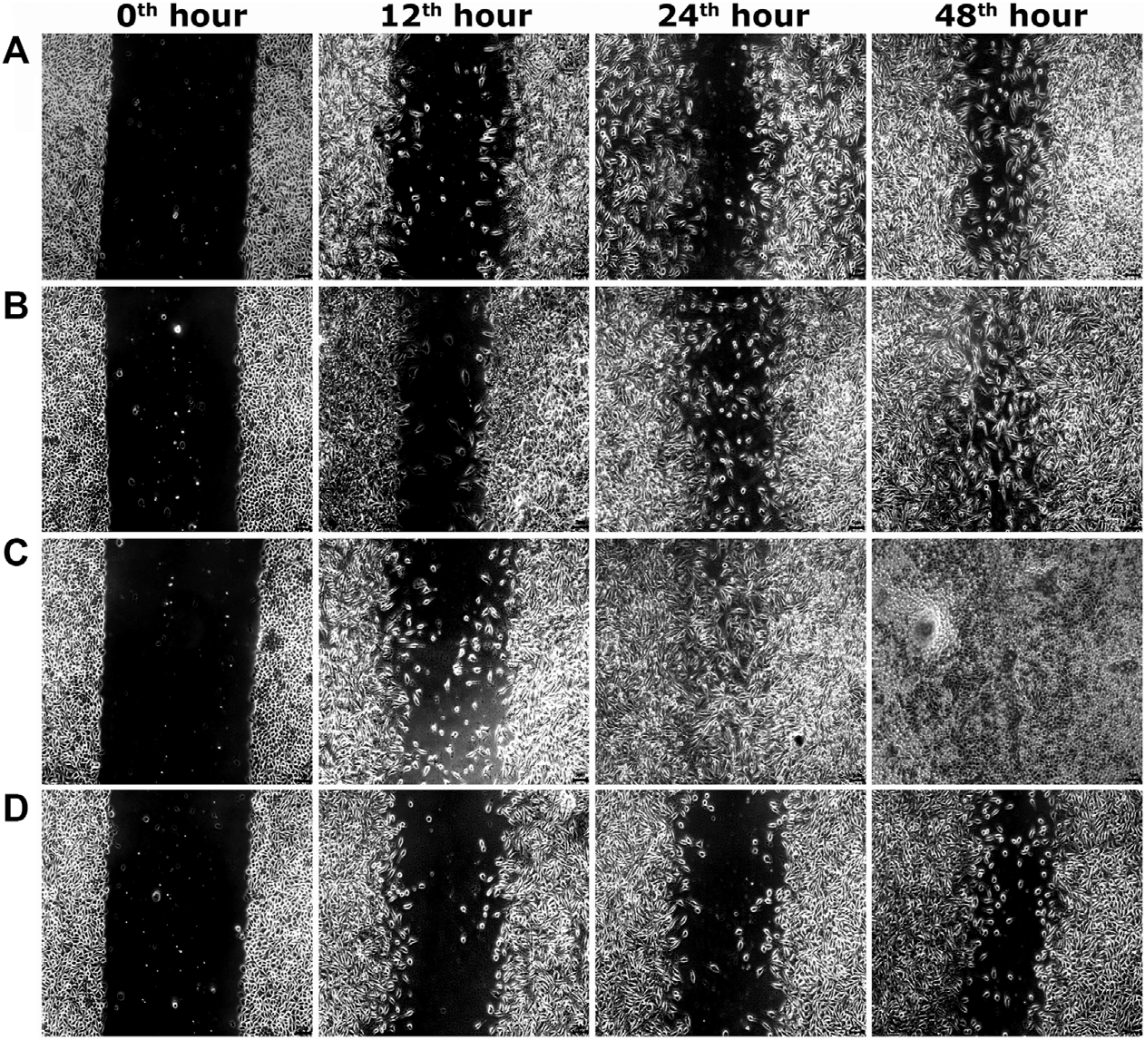
Representative images of scratch wound assay in L929 cells. **(A)** PEF at 3.90 μg/ml, **(B)** PEF at 7.81 μg/ml, **(C)** Positive control EGF at 0.002 μg/ml, **(D)** Negative control, at different post wounding hours. The original magnification: ×100 and the scale bars represent 5 µm size.

**FIGURE 3:**
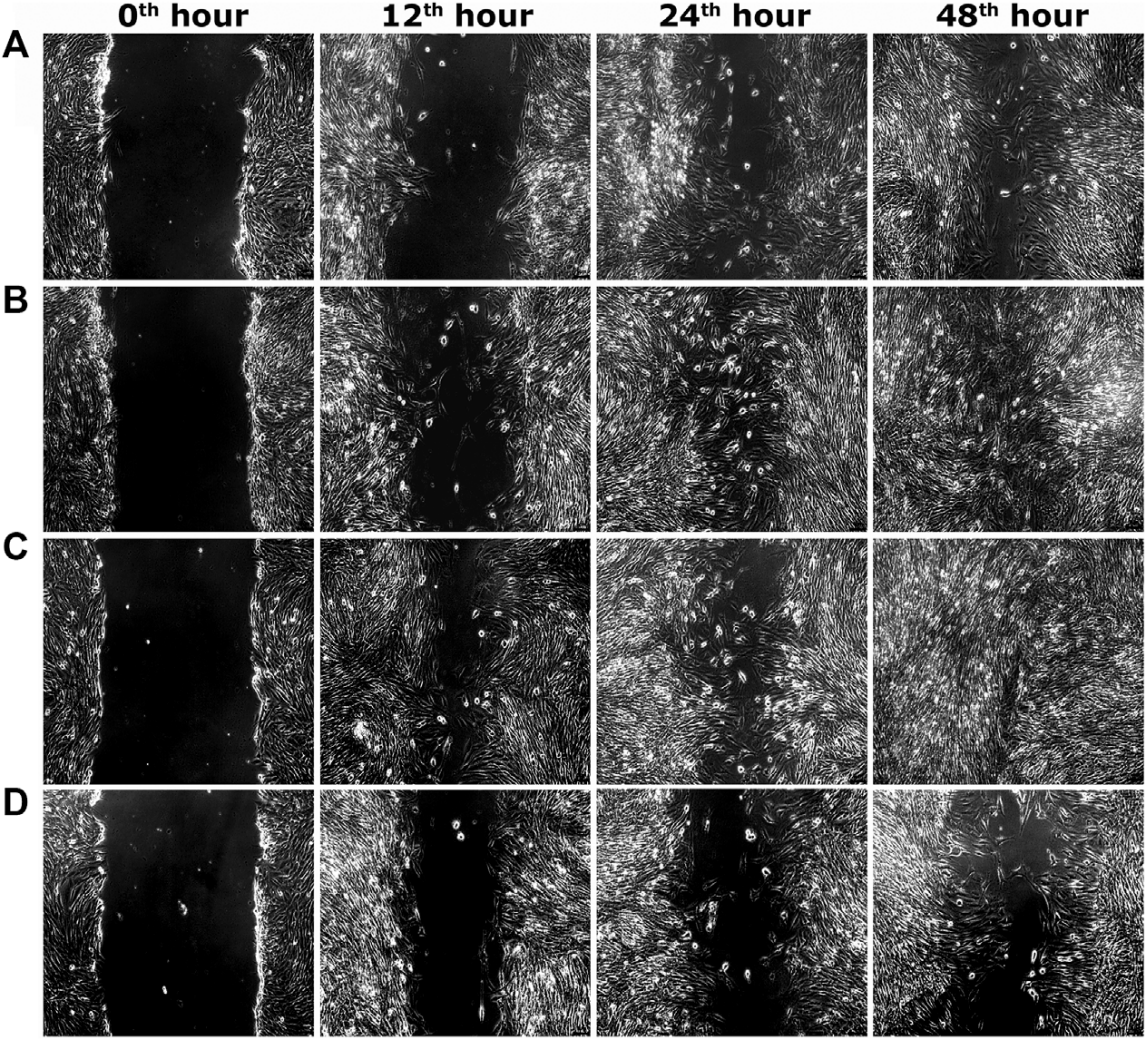
Representative images of scratch wound assay in 3T3L1 cells. **(A)** PEF at 3.90 μg/ml, **(B)** PEF at 7.81 μg/ml, **(C)** Positive control (EGF) at 0.002 μg/ml, **(D)** Negative control, at different post wounding hours. The original magnification: ×100 and the scale bars represent 5 µm size.

**FIGURE 4:**
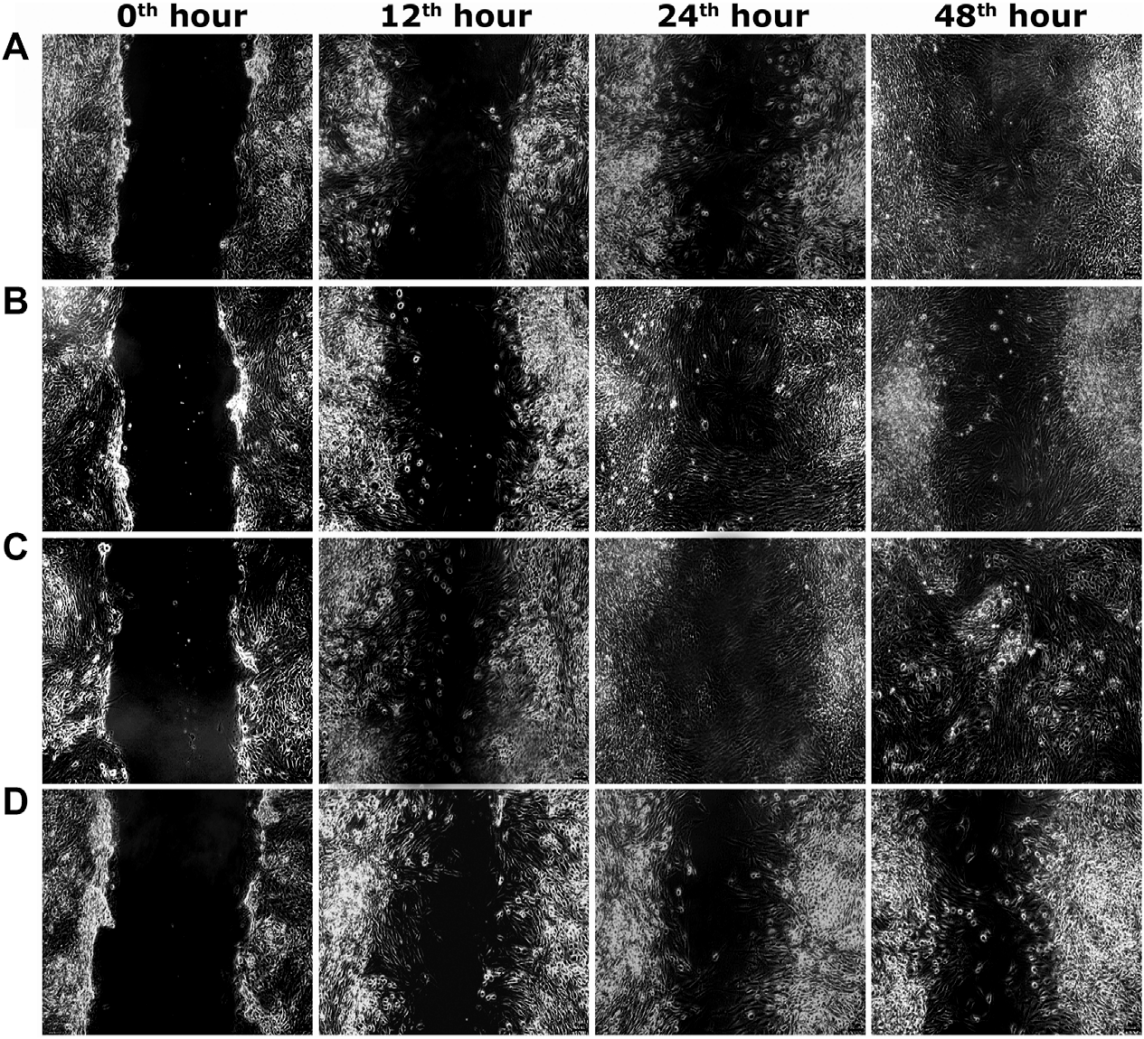
Representative images of scratch wound assay in L6 cells. **(A)** PEF at 3.90 μg/ml, **(B)** PEF at 7.81 μg/ml, **(C)** Positive control EGF at 0.002 μg/ml, **(D)** Negative control, at different post wounding hours. The original magnification: ×100 and the scale bars represent 5 µm size.

Two optimum concentrations of PEF (3.90 and 7.81 μg/ml) were preferred for *in vitro* wound scratch assay based on cell viability test. The effect of positive and negative controls and different concentrations of PEF on the migration rate of L929, 3T3L1 and L6 cells were tested up to 48 h and critically analyzed through percentage wound contraction (Figure 1. Figures 4A,B,C). The percentage of wound contraction in control group was 81.50 ± 4.00%, 77.21 ± 1.83% and 77.27 ± 4.96% for L929, 3T3L1 and L6 respectively after 48 h. PEF exhibited significant dose dependent increase in the migration of cell lines compared to control group. In L929 the migration rate was almost similar at 3.90 and 7.81 μg/ml doses with 89.16 ± 1.98% and 92.59 ± 1.53% cell migration rate at 48th hour (Figure 1A). Whereas in 3T3L1 some dose dependent variation with 91.41 ± 2.15% and 98.42 ± 0.82% cell migration rates at 3.90 and 7.81 μg/ml PEF doses respectively could be found (Figure 1B). Similar is the case with L6 cells, in which 3.90 μg/ml dose exhibits 86.65 ± 3.16% migration and it was 96.63 ± 0.61% for 7.81 μg/ml dose (Figure 1C). In EGF-treated positive control group, the migration rate of L929, 3T3L1 and L6 cells were increased to 99.39 ± 0.32%, 98.43 ± 0.80% and 98.03 ± 1.02% respectively at 48th hour of incubation period. Representative images of cell migration and wound closure are presented in Figures 2–4.

### 3.8 *In vivo* acute dermal toxicity studies

Acute toxicity study did not show any sign of toxic symptoms in both PEF and vehicle control groups at the limit test dose of 2000 mg/kg. There was no sign of any toxicity such as changes in fur, eyes, mucous membranes or any other undesirable changes on rat skin such as redness, swelling, irritation, itching and other behavioral patterns. No mortality was detected throughout the study period.

### 3.9 *In vivo* wound healing activity

**FIGURE 5:**
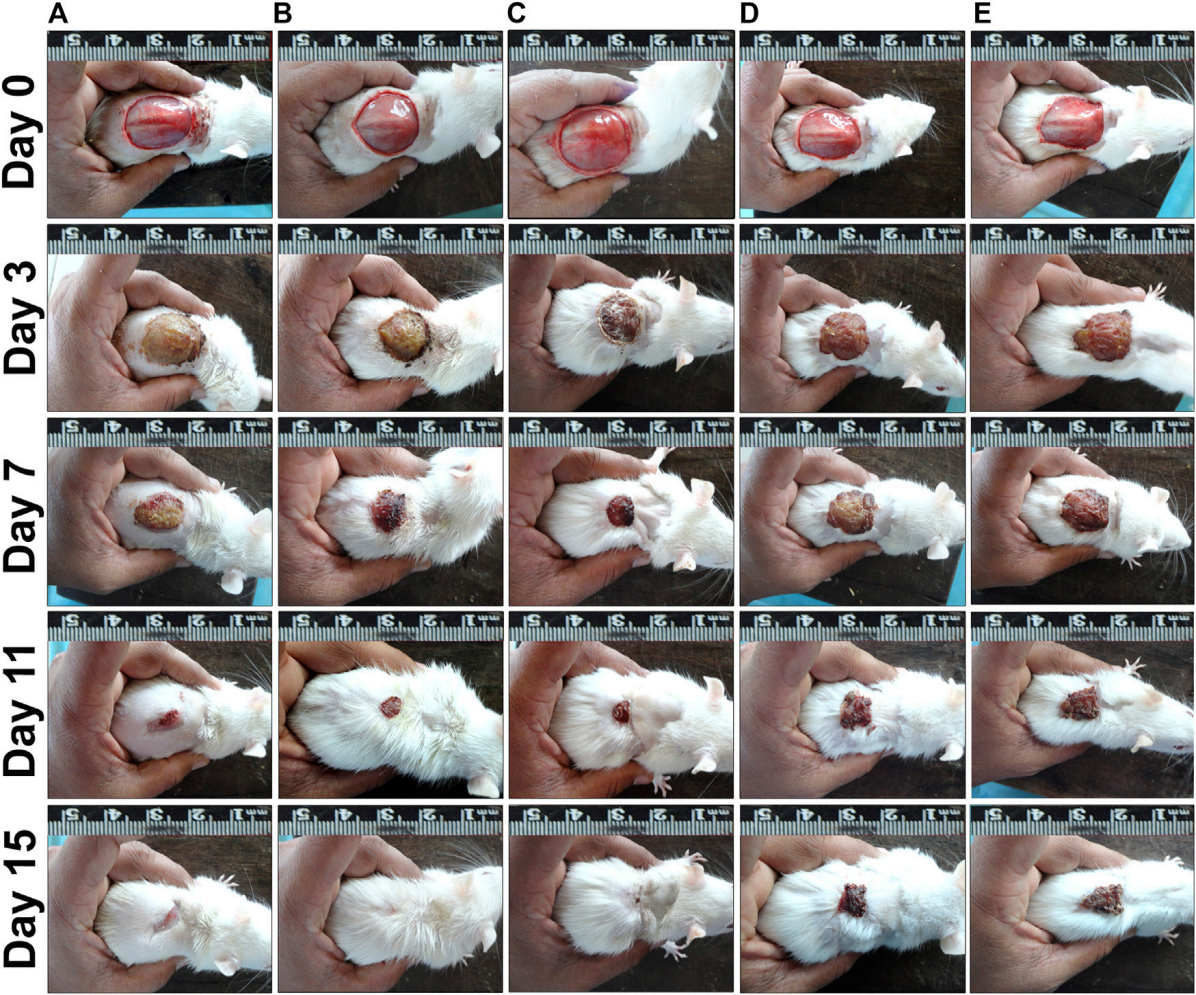
Wound contraction results on different post wounding days. **(A)** PEP 2.5% treated group, **(B)** PEP 5% treated group, **(C)** Positive control group, **(D)** Ointment base group, **(E)** Negative control group.

Rate of wound contraction in circular excision wound model are given in Table 4. PEF treated groups at 2.5% and 5% doses started showing significant results (*p* ≤ 0.05; *p* ≤ 0.01 and *p* ≤ 0.001) from seventh post wounding day compared to ointment base and negative control groups. On 15th post wounding day 2.5% and 5% PEF treatment groups showed more or less similar results (97.30 ± 0.52% and 97.92 ± 0.41% respectively) with significant results (*p* ≤ 0.05 and *p* ≤ 0.01) compared to ointment base and negative control groups. It was of interest to note that both 2.5% and 5% PEF ointment treated groups showed higher wound contraction rates without any significant differences compared to positive control group (93.67 ± 2.40%) on 15th post wounding day. Moreover, significantly less complete epithelialization period (CEP, 100% wound closure) was noticed in 5% PEF ointment (15.50 ± 0.29 days), positive control (16.00 ± 0.41 days) and 2.5% PEF ointment (16.00 ± 0.48 days) treated groups compared to ointment base and negative control groups (22.00 ± 0.41 and 23.25 ± 0.48 days respectively). The macroscopic changes of the wound healing between 0^th^ and 15th day are shown in Figure 5.

**TABLE 4:** Effect of test ointments on circular excision wound model.

	Percentage wound contraction (Mean ± S.E)				
Days	PEF 2.5%	PEF 5%	Positive control	Ointment base	Negative control
Day 1	0.20 ± 8.02	0.21 ± 1.82	4.09 ± 1.69	4.23 ± 2.87	0.44 ± 2.24
Day 3	17.78 ± 5.73	25.29 ± 4.79	14.53 ± 3.33	13.46 ± 3.12	5.98 ± 3.74
Day 5	32.90 ± 4.24	47.12 ± 2.28^a***b**c***^	18.69 ± 4.16	19.64 ± 3.46	20.97 ± 4.37
Day 7	57.71 ± 1.98^a***b*c*^	70.26 ± 2.61^a***b***c***^	34.11 ± 5.11	25.67 ± 4.61	32.10 ± 6.47
Day 9	75.88 ± 2.02^a**b**c*^	81.84 ± 1.72^a***b***c**^	49.81 ± 6.87	39.95 ± 4.15	41.25 ± 8.38
Day 11	90.31 ± 0.51^a**b**^	93.46 ± 0.85^a***b**c*^	71.68 ± 3.83	59.39 ± 5.81	62.50 ± 7.78
Day 13	94.39 ± 0.73^a***b***^	96.90 ± 0.30^a***b***^	87.95 ± 3.04^a**b**^	71.31 ± 2.66	71.17 ± 4.58
Day 15	97.30 ± 0.52^a*b**^	97.92 ± 0.41^a**b**^	93.67 ± 2.40^b*^	81.46 ± 2.92	77.37 ± 5.39
CEP in days (n = 3)	16.00 ± 0.48^a***b***^	15.50 ± 0.29^a***b***^	16.00 ± 0.41^a***b***^	22.00 ± 0.41	23.25 ± 0.48

S.E, standard error; CEP, Complete epithelialization period. Significant differences at ^*^ *p* ≤ 0.05; ^**^ *p* ≤ 0.01; ^***^ *p* ≤ 0.001 were noted between the variables of treatment groups at different post wounding days. Analysis of the data was conducted using one-way ANOVA with Tukey post hoc test. Different letters (^a,b,c^) in each column exhibits significant difference against ointment base, negative control and positive control groups respectively.

The results of tensile strength of incised wounds are shown in Table 5. Of the tested concentrations, 5% PEF ointment treated group showed maximum tensile strength (973.67 ± 4.43 g/mm^2^) with highly significant differences (*p* ≤ 0.001) against negative control, ointment base, positive control and 2.5% PEF ointment treated groups. Interestingly in the incised wounds, both 2.5% and 5% PEF ointment treated groups showed relatively higher tensile strength compared to positive control group on 15th post wounding day (Table 5).

**TABLE 5:** Effect of test ointments on paravertebral incision wound model.

Treatment groups	Tensile strength (g/mm^2^) (Mean ± S.E)
PEF 2.5%	860.45 ± 3.04^a***,b***,c*^
PEF 5%	973.67 ± 4.43^a***,b***,c*** ,d***^
Positive control	814.02 ± 3.59^a***,b***^
Ointment base	536.78 ± 14.78
Negative control	524.30 ± 11.34

S.E: standard error; CEP: Complete epithelialization period. Significant differences at*p ≤ 0.05; **p ≤ 0.01; ^***^ *p* ≤ 0.001 were noted between the variables of treatment groups at different post wounding days. Analysis of the data was conducted using one-way ANOVA with Tukey post hoc test. Different letters (^a,b,c,d^) in each column exhibits significant difference against ointment base, negative control, positive control and PEF 2.5% treated groups respectively.

**FIGURE 6:**
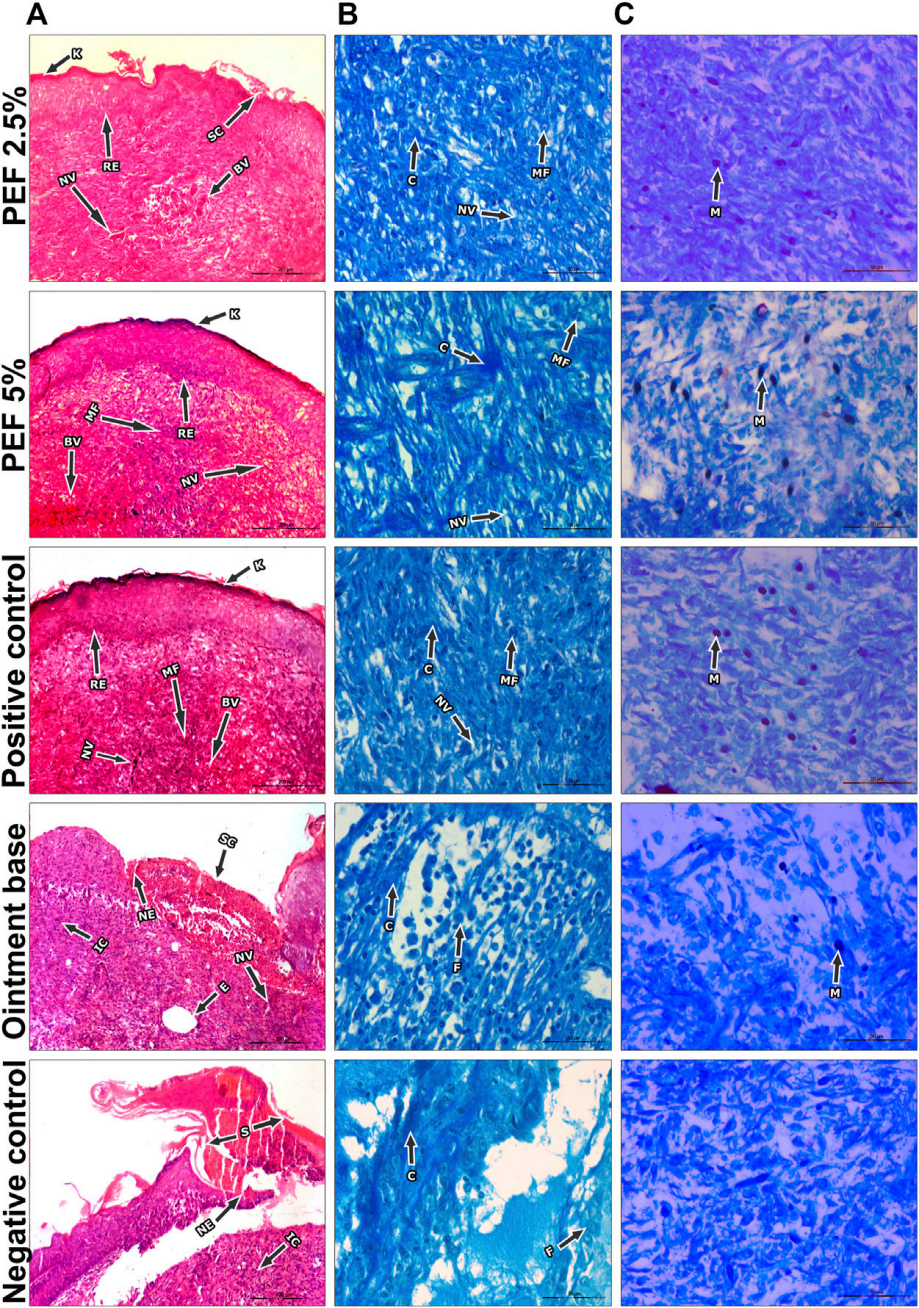
Histopathological view of excision wound model. **(A)** H&E stained sections, **(B)** Masson’s trichome stained sections, **(C)** Toluidine blue stained sections in corresponding treatement groups. Original magnification was ×100 in H&E stained sections and the scale bars signify 200 µm. The magnification ×400 for Masson’s Trichome and Toluidine blue stained sections and the scale bars signify 20 µm. Arrows representing different cell components in the sections are RE: Re-epithelization, (K) Keratinization, ET: Epidermal thickness, BV: Blood vessels, SC, Scab, (C) Collagen, NV:Neovascularization, IC: Inflammatory cells, NE: Necrosis, (E) Edema, MF, Myofibroblasts, MA, Macrophages, BV, Blood vessels, N, Neutrophils, MC, Mast cells.

On 15th post wounding day skin tissues of the animals from all the treatment groups were taken for histopathological examination (Figure 6). H&E stained images revealed greater tissue regeneration in 5% PEF, positive control and 2.5% PEF treated groups with re-epithelialization of granular tissues, dense collagen fibers, formation of epidermal layer and keratinization. It was also supported by the Masson’s trichome stained images, in which the intense blue color corresponds to the relatively higher quantity of the collagen fiber deposit in 5% PEF treated group. However, from the comparative histological scoring it was observed that 5% PEF and positive control groups showed higher scoring patterns in terms of re-epithelization, epidermal thickness, keratinization, collagen deposition and neovasularization (Table 6). Similar pattern was also observed in differential cell count results, in which these two groups (5% PEF and positive control groups) showed more number of myofibroblasts, mast cells and newly formed blood vessels (angiogenesis). Significant reduction in the neutrophils, macrophages and inflammatory cells demonstrated the efficacy of the treated drugs in complete regeneration and matrix remodeling of the wounded tissues (Table 7). In contrast to these, in ointment base and negative control groups lack of epidermal layer formation, infiltration of exudates and fibrinoid necrosis in subepidermal region indicated poor matrix organization and re-epithelialization. Empty spaces in the dermal region indicated the evidence of edema; increased neutrophils, macrophages and inflammatory cell counts suggested the incomplete healing process.

**TABLE 6:** Histopathological evaluation of animal skin sections corresponding to different treatment groups.

Treatment groups	Parameters							
	RE	ET	K	NE	E	SC	C	NV
PEF 2.5%	**++**	**++**	**+++**	**-**	**-**	**+**	**++**	**+**
PEF 5%	**+++**	**+++**	**+++**	**-**	**-**	**-**	**+++**	**++**
Positive control	**+++**	**+++**	**+++**	**-**	**-**	**-**	**+++**	**++**
Ointment base	**-**	**-**	**-**	**++**	**++**	**++**	**+**	**+**
Negative control	**-**	**-**	**-**	**+++**	**+++**	**+++**	**+**	**+**

Scoring pattern represents: ‘-’ absent, ‘+’ mild, ‘++’ moderate and ‘+++’ intense. RE, Re-epithelization; ET, epidermal thickness; K, keratinization; NE, necrosis; E, edema; SC, scab; C, collagen; NV, neovascularization.

**TABLE 7:** Quantification of cells in the granulation tissue sections of treated animal groups.

Treatment groups	Cell counts in different magnifications of stained sections					
	IC (100×)	MF (400×)	MA (400×)	BV (100×)	N (400×)	MC (400×)
PEF 2.5%	92.67 ± 3.48^a^	10.00 ± 1.53^a^	10.67 ± 0.88^a^	22.67 ± 1.45^a^	13.33 ± 0.67^a^	6.00 ± 1.15^a^
PEF 5%	68.33 ± 2.03^b^	17.33 ± 0.88^b^	5.67 ± 0.88^b^	28.67 ± 1.20^b^	7.67 ± 0.88^b^	11.33 ± 0.88^b^
Positive control	59.67 ± 4.63^b^	21.33 ± 1.45^b^	3.00 ± 0.58^b^	34.67 ± 0.88^c^	4.67 ± 0.88^c^	15.67 ± 0.88^c^
Ointment base	132.33 ± 4.70^c^	7.67 ± 0.33^a,c^	15.67 ± 0.88^c^	11.67 ± 0.88^d^	19.33 ± 2.03^c^	3.00 ± 0.58^a^
Negative control	164.33 ± 4.05^d^	6.33 ± 0.88^a,c^	28.67 ± 1.20^d^	7.67 ± 0.88^d^	25.67 ± 0.88^d^	2.67 ± 0.33^a^

IC, inflammatory cells; MF, myofibroblasts; MA, macrophages; BV, blood vessels; N, neutrophils; MC, Mast cells. Values are expressed as mean ± standard error. Different letters (^a,b,c,d^) in each column represent significant differences between the variables of associated groups through Tukey’s Post hoc analysis at *p* ≤ 0.05.

**FIGURE 7:**
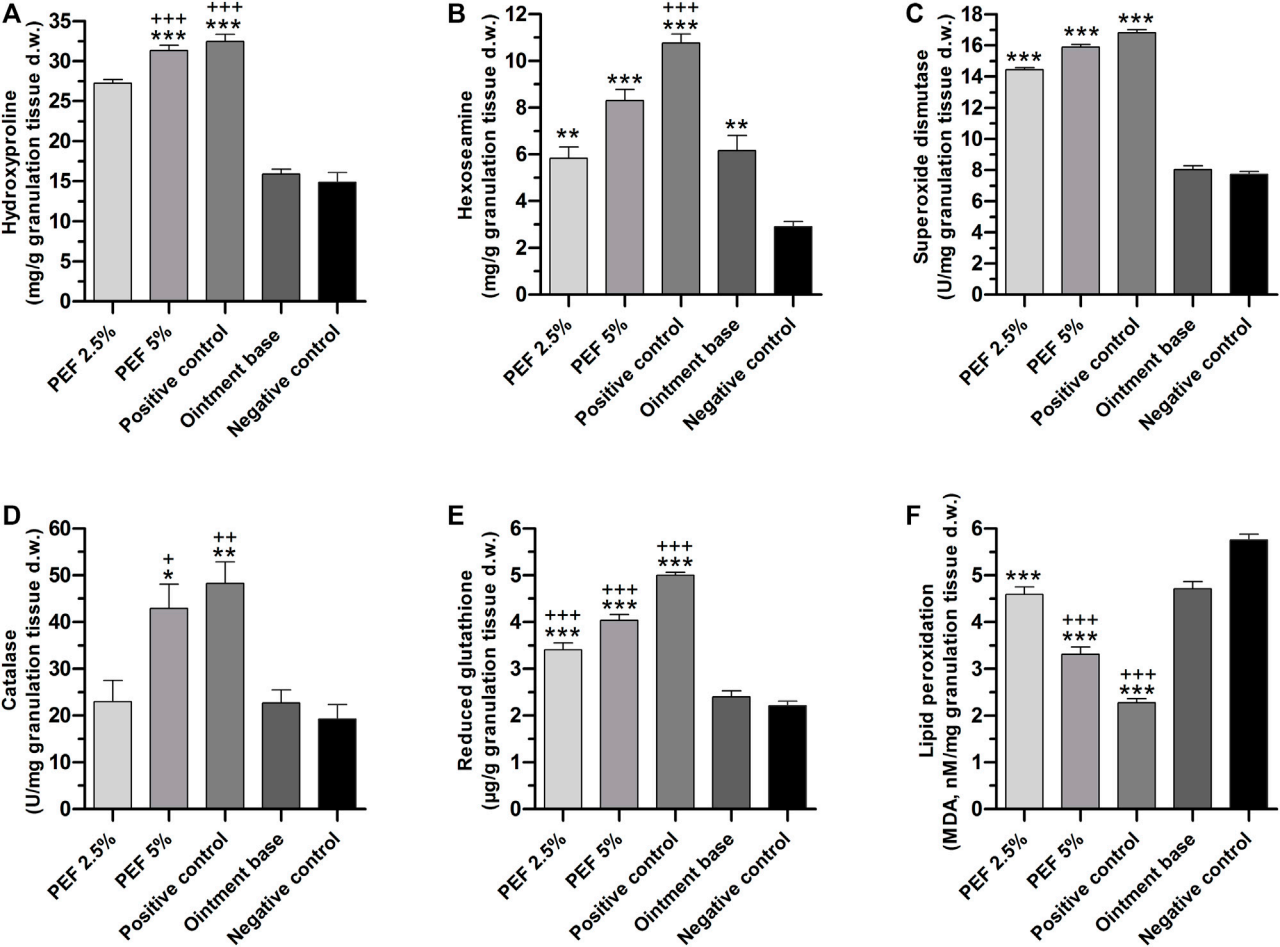
Biochemical parameters of granulation tissues in different treatment groups. **(A)** Hydroxyproline, **(B)** Hexoseamine, **(C)** SOD, **(D)** Catalase, **(E)** GSH, **(F)** LPO. “*” indicates significant differences (*p* ≤ 0.05, *p* ≤ 0.01 or *p* ≤ 0.001) between treatment groups and negative control group. “+” indicates significant differences (*p* ≤ 0.05, *p* ≤ 0.01or *p* ≤ 0.001) between treatment groups and ointment base treated group. Analysis of the data was conducted using one-way ANOVA with Tukey *post hoc test*.

In the process of wound healing, hydroxyproline and hexoseamine are considered as important elements due to their crucial role in the formation of collagen fibers. In the present study, these connective tissue parameters were measured in granulation tissue of all the treatment groups (Figures 7A,B). It was found that 5% PEF and positive control treated groups showed significantly higher quantity (*p* ≤ 0.001) of hydroxyproline with 31.31 ± 0.64 and 32.44 ± 0.89 mg/g compared to ointment base and negative control groups. Similarly, hexosamine contents of positive control (10.77 ± 0.38 mg/g) as well as 5% and 2.5% PEF treated groups (8.30 ± 0.47 and 5.83 ± 0.48 mg/g respectively) showed significant results in comparison with ointment base and negative control groups.

Among the enzymatic antioxidant parameters, SOD levels in the granulation tissues of positive control (16.81 ± 0.18 U/mg), 5% PEF (15.89 ± 0.17 U/mg) and 2.5% PEF (14.43 ± 0.14 U/mg) treated groups were significantly high (*p* ≤ 0.001) compared to ointment base and negative control groups (8.03 ± 0.24 and 7.72 ± 0.20 U/mg respectively) (Figure 7C). Meanwhile, the quantity of CAT enzyme was also significantly increased in positive control and 5% PEF treated groups (48.30 ± 4.60 and 42.91 ± 5.18 U/mg respectively) (Figure 7D).

The non-enzymatic antioxidant parameter GSH was significantly increased in positive control (5.00 ± 0.06 μg/g) and in 5% and 2.5% PEF treated groups (4.04 ± 0.12 and 3.41 ± 0.15 μg/g respectively) against negative control and ointment base treated groups with *p* ≤ 0.001 (Figure 7E).

However, LPO, the oxidative stress mediated marker, was significantly lowered (*p* ≤ 0.001) in positive control, 5% and 2.5% PEF treated groups compared to negative control and ointment base treated groups (Figure 7F). Apparently, the lowering of LPO content is a clear indication of active role of PEF and positive control (Povidone-Iodine) in preventing the oxidative damage and inflammation, which indeed promotes the healing process.

### 3.10 GC-MS analysis

Altogether eight compounds were identified through GC-MS analysis of PEF based on their mass spectra and retention indices. The name of compounds with their retention time, peak area percentage, molecular formula, molecular weight, compound nature, and Kovats retention index of each compound are given in Table 8. The major phenolic compounds were found to be gallic acid and ethyl gallate with the peak area 47.23% and 35.52% respectively.

**TABLE 8:** Chemical composition of PEF analyzed through GC-MS instrument.

Retention time (in min)	Name of the compound	Molecular formula	Kovats retention index	Molecular weight (g/mol)	Compound nature	Peak area %
12.243	Gallic acid	C_6_H_6_O_3_	1754	170.12	Tannin	47.23
12.557	Pyrogallol	C_6_H_6_O_3_	1,329	126.11	Tannin	2.32
17.641	Palmitic acid methyl ester	C_17_H_34_O_2_	1908	274.196	Fatty acid methyl ester	1.47
18.164	Palmitic acid	C_16_H_32_O_2_	1942	256.42	Fatty acid	3.10
19.191	Ethyl gallate	C_9_H_10_O_5_	1922	198.17	Tannin	35.52
19.584	Methyl stearate	C_19_H_38_O_2_	2,114	298.5	Fatty acid	1.60
19.851	Oleic acid	C_18_H_34_O_2_	2098	282.5	Fatty acid	2.39
20.065	Stearic acid	C_18_H_36_O_2_	2,187	284.5	Fatty acid	6.36

### 3.11 RP-UFLC-DAD analysis

The result obtained from GC-MS analysis revealed the presence of gallic acid and ethyl gallate as major phenolic compounds. This prompted to carry out RP-UFLC-DAD analysis to find out the concentration of these compounds in PEF (Supplementary Figures S4A, S4B). Identification of sample peaks were confirmed by comparing UV absorption spectra (271 nm) and retention time of standard gallic acid (3.145 ± 0.042 min) and ethyl gallate (16.089 ± 0.027 min) respectively. High linearity responses and correlation coefficients (*R* ^2^ = 0.999) were observed through calibration curve equation drawn from eight different concentrations of standard gallic acid and ethyl gallate (Supplementary Figures S4C, S4D). The LOD and LOQ of gallic acid were 0.04 and 0.11 μg/ml, whereas it was 0.02 and 0.07 μg/ml for ethyl gallate. The methods were found precise and reproducible as indicated by less than 2% relative standard deviation (RSD) values. Validation parameters were executed by spiking the volume of 50 μL (5 μg/ml) of standards with equal volume of PEF and the recovery was obtained within the range of 95–100%. The quantity of gallic acid and ethyl gallate in PEF were 68.08 μg/mg and 255.91 μg/mg respectively.

## 4 Discussion

In spite of availability of advanced healthcare in the modern medicine, herbal products and their crude preparations, derived from ethnomedicinal plants are still in great demand in the treatment of certain diseases. Wound management is one such widespread condition for which many are dependent on plants due to their easy availability, proven effectiveness and apparently fewer side effects (Agyare et al., 2009; Cragg and Newman, 2013). In our previous work we evaluated wound healing efficacy of crude ethanol extract of *C. mimosoides*, an indigenous medicinal plant widely known for its wound healing activity (Bhat et al., 2016). It is noted that crude extract contains diverse phytoconstituents and efficacy of crude extract could be because of their synergistic activities. However, enrichment process limits to specific quantifiable fractions with measurable pharmacological activity (Azis et al., 2017). Similar wound healing efficacy of different phenol enriched fractions from crude extracts is also reported by earlier workers in various other plant species (Ruiz-Ruiz et al., 2017; Yadav et al., 2017; Yadav et al., 2018; Xavier-Santos et al., 2022).

Earlier studies on *Prosopis cineraria* (Yadav et al., 2018), Kigelia africana (Lam.) Beneth. and *Strophanthus hispidus* DC (Agyare et al., 2013). and *Entada africana* Guill. & Perr (Baidoo et al., 2021). have reported the use of plant extracts enriched with phenolic compounds accelerate the wound healing activity due to their higher antioxidant, anti-inflammatory and antimicrobial properties. Similar investigation on *C. mimosoides* PEF indicated expressive amounts of phenolic and flavonoid contents (578.28 ± 2.30 mg GAE/g and 270.76 ± 2.52 mg QE/g) and the ointment of PEF containing 2.5% and 5% dose demonstrated effective pro-healing efficacy in both excision and incision wound models, when topically applied for 15 days. The GC-MS analysis of PEF displayed various chemical constituents, predominantly occupied by gallic acid and ethyl gallate and it is also confirmed by RP-UFLC-DAD analysis with 68.08 and 255.91 μg/mg concentrations respectively. The reactive oxygen species (ROS) synthesized at the wound site, play a crucial role as secondary messengers and provides defense against microbial infection. They also regulate vasoconstriction and vasodilation of the normal wound healing process; however, it’s over production at the wound site produce oxidative stress and that results delay in wound healing (Dunnill et al., 2017). Antioxidants are the agents that convert ROS into more stable molecules through complex cascade of reactions and protect the cells from being damaged by these free radicals. It has been attributed that the antioxidant activities of plant extracts are mainly due to high phenolic and flavonoid content and they are positively correlated with wound healing efficacy (Agyare et al., 2013; Vittorazzi et al., 2016; Baidoo et al., 2021). Our results suggest that the significant antioxidant property of PEF might have contributed to a great extent in the observed wound healing activity.

Protein denaturation is the process in which the proteins lose their secondary and tertiary structures caused by noxious external stress, injuries, chemicals or other agents. Most of the biological proteins lose their functions when they get denatured. Protein denaturation in tissues is mainly due to autoantigens in certain inflammatory and arthritic diseases (Ruiz-Ruiz et al., 2017). Similarly, the proteinase enzymes can promote inflammation by regulating the expression of pro-inflammatory cytokines, chemokines and other immune components at the site of cause (Chakraborty and Bhattacharyya, 2013). Results revealed that PEF exhibited strong *in vitro* anti-inflammatory activity by inhibiting both protein denaturation and proteinase activity in dose dependent manner, similar to standard diclofenac sodium. Inhibition activity might have provided the protection from tissue damage complications.

Open wounds are prone to microbial attack and thus make them susceptible to develop complications of infections. Microorganisms produce harmful effects on the host organism by causing increased pain, distress and thereby significantly impair the normal wound healing process (Houghton et al., 2005; Misic et al., 2014; Vittorazzi et al., 2016). The most common bacterial and fungal pathogens usually invade the wounds and cause other skin related problems are *S. typhi* and *K. pneumoniae* (skin lesions); *S. aureus*, *P. aeruginosa* and *P. vulgaris* (wound infections); *M. luteus* and *M. flavus* (intracranial abscesses); *C. albicans* (candidiasis); *M. furfur* (dermatitis, folliculitis etc.) (Bhat et al., 2016). Effective antimicrobial agents enhance the wound healing process in one or more phases by forming a barrier against infectious microbes. Therefore, the topical application of active antimicrobial agents is one of the efficient wound healing therapies to alleviate the possible wound infections (Suntar et al., 2013; Baidoo et al., 2021). In the present study PEF showed broad spectrum of antimicrobial activity against microbial skin pathogens with significant MIC and ZI, even more than crude ethanol extract. In this context, it could be inferred that the topical application of ointment containing PEF protects the cutaneous wounds from pathogens and thereby supports the efficient wound healing process.

Cell viability or cytotoxicity was evaluated to determine the dose response of the cells against treatment drugs. It also provides valuable information about the safe dose of active principles in the test drugs, when topically applied (Silva et al., 2019). This is to find out the effects of metabolites on basic functions of cells or skin tissues which help to judge whether they can be considered for evaluating biological activity or not. American National Cancer Institute (ANCI) have standardized the IC_50_ of extracts to 30 μg/ml and considered it as toxic for cells (Bolla et al., 2019). In our study IC_50_ of PEF against all the three cell lines were >250 μg/ml, which is predominantly higher than the recommended thresholds set by ANCI. More than 90% of cell viability was reported at ≤7.81 μg/ml dose of PEF for L6 and ≤15.62 μg/ml for both L929 and 3T3L1 cells respectively. This safety margin prompted to carry out *in vitro* wound scratch assay of PEF within their safe doses.

*In vitro* wound scratch study proved that PEF effectively stimulated the cell migration rates in L929 (89.16 ± 1.98% and 92.59 ± 1.53%), irrespective of two different concentrations (3.90 μg/ml and 7.81 μg/ml) at 48th hour of incubation. However, in 3T3L1 and L6 cell lines the cell migration rate increased significantly in a dose dependent manner. A similar study on two phenolic compounds (chlorogenic acid and myricetin-3-O-β-rhamnoside) isolated from Parrotia persica (DC.) C.A.May reported that the compounds have effectively stimulated the cell migration rate of HUVEC fibroblast cells at 10 μg/ml dose (Moghadam et al., 2017). Further, the present study is in agreement with the study conducted by Azis et al. (2017), wherein the asiaticoside enriched methanol fraction from *Centella asiatica* showed significant dose dependent effect on HDF and HaCaT cells at 0.2 μg/ml and 100 μg/ml concentrations respectively.

A limit test dose of PEF at 2000 mg/kg did not show any toxic symptoms in the treated animals. The results of *in vivo* circular excision and paravertebral incision wound studies showed enhanced rate of wound contraction with the treatment of PEF at 2.5% and 5% doses on 15th post wounding day. However, significantly shorter CEP (100% wound closure) was noticed in 5% PEF (15.50 ± 0.29 days), even better than that of standard Povidone-Iodine ointment treated group (Table 4). The ointment base and negative control groups took longer time (more than 21 days) for complete epithelialization. Concurrently, the efficiency of 5% PEF was also reflected in incised wounds with significant higher tensile strength compared to other groups (Table 5). It is opined that the wound re-epithelization in a shorter period is a hallmark of successful wound care and the same in PEF treated groups is possibly due to its efficiency in facilitating the viability and proliferation of epithelial cells, followed by increased keratinocyte proliferation to the wound cite at a faster rate than other groups (Mekonnen et al., 2013).

The contraction and tensile strength of incised wounds mainly depends on the accumulation and stabilization of collagen fiber contents at the wound site (Murthy et al., 2013). Collagen is one of the important proteins in the extracellular matrix, liberates free hydroxyproline and its peptides. Hence, measurement of hydroxyproline contents in the granulation tissue is a key index for collagen turnover. Additionally, hexosamine contents in the granulation tissue reflect the stabilization of collagen molecules by enhancing electrostatic and ionic interactions (Dwivedi et al., 2016). Increased hydroxyproline and hexosamine contents are positively correlated to collagen synthesis and thereby enhanced wound healing (Gurung and Skalko-Basnet, 2009; Dwivedi et al., 2016). In the present study higher collagen content in 5% PEF treated group might have played a crucial role in rapid wound healing with shorter epithelialization period. It is evidenced by increased hydroxyproline and hexosamine contents (31.31 ± 0.64 and 8.30 ± 0.47 mg/g) in the PEF treated animal tissues. Further, a clear differentiation of dose dependent activity of PEF was also detected in histopathological examination of granulation tissues. In H&E stained sections 5% PEF treated and positive control groups showed complete epidermal regeneration and formation of keratin layers. The granular layer was found to be well formed with 2-3 cells in thickness with maximum cellular infiltration, well oriented collagen fibers, more proliferation of myofibroblasts, prominent capillary-sized blood vessels, scattered distribution of inflammatory cells, less in number of macrophages and neutrophils. The transformation of fibroblasts to myofibroblasts during formation of granulation tissue is the indicating factor of wound contraction (Ponrasu et al., 2013).

Wound undergoes substantial oxidative stress during healing process by overproduction of neutrophils-derived ROS. It has been demonstrated that cells synthesize variety of enzymatic and non-enzymatic scavengers as an antioxidant defense mechanism. Among these, SOD plays a critical role in the attenuation of oxidative stress through dismutation of O_2_^−^. In addition, CAT enzyme has an important role in converting the endogenous hydrogen peroxide to water and oxygen and thereby prevents the cells from oxidative damages. Another important antioxidant enzyme, GSH reduces the peroxide radicals by accelerating Glutathione peroxidase enzyme (Moghadamtousi et al., 2015). Results obtained from the present investigation indicated that the administration of 5% PEF in the treatment groups lead to a significant upregulation of these three enzymes (SOD, CAT and GST) and exponential reduction in the levels of oxidative stress (LPO) in granulation tissues. These findings are at par with the results of earlier studies, where the efficacy of protocatechuic acid rich n-butanol fraction of *Trianthema portulacastrum* and phenol enriched fraction of Prosopis cineraria in enhancing the cellular antioxidant activities is established (Yadav et al., 2017; Yadav et al., 2018). Hence it could be deduced that the phenolic compounds present in PEF contribute to its elevated cellular antioxidant activity.

## 5 Conclusion

The study established the efficacy of ointment containing PEF of *C. mimosoides* in accelerating the wound healing process and eventually shortening the healing period. The study also established the positive correlations between antimicrobial, antioxidant and anti-inflammatory activities in wound healing, and the presence of phenolic compounds such as ethyl gallate, gallic acid and pyrogallol that could be contributing in enhancing the wound healing activity. The study makes a case for further bioactivity guided isolation of active constituents in PEF for elucidating the mechanism of action of individual phytocompounds and for further drug development.

## Data Availability

The original contributions presented in the study are included in the article/Supplementary Material, further inquiries can be directed to the corresponding author.

## Glossary

ANCI: American National Cancer Institute
ANOVA: Analysis of variance
BDS: Base Deactivated Silica
BHA: butylated hydroxyanisole
CAT: catalase
CEP: Complete epithelization period
CFU: Colony forming units
dw: dry weight
DAD: diode array detector
DMEM: Dulbecco’s Modified Eagle’s Medium
DMSO: Dimethyl sulfoxide
DPPH: 2,2-diphenyl-1-picrylhydrazyl
EGF: epidermal growth factor
FBS: fetal bovine serum
GC-MS: Gas Chromatography-Mass spectroscopy
GSH: reduced glutathione
H&E: hematoxylin & eosin stain
ICH: International Council on Harminisation
IP: Indian Pharmacopoeia
LC: Liquid chromatography
LOD: Limit of detection
LOQ: Limit of quantification
LPO: lipid peroxidation
MIC: Minimum inhibitory concentration
MTCC: Microbial Type Culture and Collection
MTT: (3-[4,5-Dimethylthiazol-2-yl]-2,5- diphenyltetrazolium bromide)
NCIM: National Collection of Industrial Microorganisms
NCCS: National Centre for Cell Science
OECD: Organisation for Economic Cooperation and Development
PBS: phosphate buffer saline
PEF: phenol enriched fraction
ROS: Reactive oxygen species
RP-UFLC-DAD: Reversed phase-ultra flow liquid chromatography-diode array detector
RSD: Relative standard deviation
SOD: superoxide dismutase
USP: United States Pharmacopoeia
UV: ultraviolet
WHO: World Health Organization
ZI: zone of inhibition.

## References

[B1] Adams R. P. (2007). Identification of essential oil components by gas chromatography/mass spectroscopy. Carol Stream (IL): Allured.

[B2] Agyare C. Asase A. Lechtenberg M. Niehues M. Deters A. Hensel A. (2009). An ethnopharmacological survey and *in vitro* confirmation of ethnopharmacological use of medicinal plants used for wound healing in Bosomtwi–Atwima–Kwanwoma area, Ghana. J. Ethnopharmacol. 125, 393–403. 10.1016/j.jep.2009.07.024 19635544

[B3] Agyare C. Dwobeng A. S. Agyepong N. Boakye Y. D. Mensah K. D. Ayande P. G. (2013). Antimicrobial, antioxidant, and wound healing properties of Kigelia africana (Lam.) Beneth. and Strophanthus hispidus DC. Adv. Pharmacol. Sci. 2013, 692613. 10.1155/2013/692613 23662099 PMC3639673

[B5] Anwar S. Almatroudi A. Allemailem K. S. Joseph R. J. Khan A. A. Rahmani A. H. (2020). Protective effects of ginger extract against glycation and oxidative stress-induced health complications: An *in vitro* study. Processes 8, 468. 10.3390/pr8040468

[B6] Arunachalam K. Parimelazhagan T. (2013). Anti-inflammatory, wound healing and *in vivo* antioxidant properties of the leaves of Ficus amplissima Smith. J. Ethnopharmacol. 145, 139–145. 10.1016/j.jep.2012.10.041 23123798

[B7] Astaneie F. Afshari M. Mojtahedi A. Mostafalou S. Zamani M. J. Larijani B. (2005). Total antioxidant capacity and levels of epidermal growth factor and nitric oxide in blood and saliva of insulin-dependent diabetic patients. Arch. Med. Res. 36, 376–381. 10.1016/j.arcmed.2005.03.007 15950078

[B8] Azis H. A. Taher M. Ahmeda A. S. Sulaiman W. M. A. W. Susanti D. Chowdhury S. R. (2017). *In vitro* and *in vivo* wound healing studies of methanolic fraction of *Centella asiatica* extract. S. Afr. J. Bot. 108, 163–174. 10.1016/j.sajb.2016.10.022

[B9] Baidoo M. F. Mensah A. Y. Ossei P. P. S. Asante-Kwatia E. Amponsah I. K. (2021). Wound healing, antimicrobial and antioxidant properties of the leaf and stem bark of Entada africana Guill. & Perr. S. Afr. J. Bot. 137, 52–59. 10.1016/j.sajb.2020.09.037

[B10] Bergman I. Loxley R. (1963). Two improved and simplified methods for the spectrophotometric determination of hydroxyproline. Anal. Chem. 35, 1961–1965. 10.1021/ac60205a053

[B11] Bhat P. B. Hegde S. Upadhya V. Hegde G. R. Habbu P. V. Mulgund G. S. (2016). Evaluation of wound healing property of *Caesalpinia mimosoides* Lam. J. Ethnopharmacol. 193, 712–724. 10.1016/j.jep.2016.10.009 27717906

[B12] Bhat P. Hegde G. Hegde G. R. (2012). Ethnomedicinal practices in different communities of Uttara Kannada district of Karnataka for treatment of wounds. J. Ethnopharmacol. 143, 501–514. 10.1016/j.jep.2012.07.003 22820243

[B13] Bhat P. Hegde G. R. Hegde G. Mulgund G. S. (2014). Ethnomedicinal plants to cure skin diseases-An account of the traditional knowledge in the coastal parts of Central Western Ghats, Karnataka, India. J. Ethnopharmacol. 151, 493–502. 10.1016/j.jep.2013.10.062 24239890

[B14] Biswas D. Yoganandam G. P. Dey A. Deb L. (2013). Evaluation of antimicrobial and wound healing potentials of ethanol extract of Wedelia biflora Linn. D.C. leaves. Indian J. Pharm. Sci. 75, 156–161. 24019563 PMC3757853

[B15] Bolla S. R. Al-Subaie A. M. Al-Jindan R. Y. Balakrishna J. P. Ravi P. K. Veeraraghavan V. P. (2019). *In vitro* wound healing potency of methanolic leaf extract of Aristolochia saccata is possibly mediated by its stimulatory effect on collagen-1 expression. Heliyon 5, e01648. 10.1016/j.heliyon.2019.e01648 31193473 PMC6529694

[B16] Brand-Williams W. Cuvelier M. E. Berset C. (1995). Use of free radical method to evaluate antioxidant activity. Leb. Wiss Technol. 28, 25–30. 10.1016/S0023-6438(95)80008-5

[B17] Brem H. Tomic-Canic M. (2007). Cellular and molecular basis of wound healing in diabetes. J. Clin. Invest. 117, 1219–1222. 10.1172/JCI32169 17476353 PMC1857239

[B18] Carvalho M. T. B. Araújo-Filho H. G. Barreto A. S. Quintans-Júnior L. J. Quintans J. S. S. Barreto R. S. S. (2021). Wound healing properties of flavonoids: A systematic review highlighting the mechanisms of action. Phytomedicine. 90, 153636. 10.1016/j.phymed.2021.153636 34333340

[B19] Chakraborty K. Bhattacharyya A. (2013). “Role of proteases in inflammatory lung diseases,”. Proteases in health and disease, advances in biochemistry in health and disease. Editors Chakraborti Dhalla N. S., 7. Springer Nature, Switzerland, 10.1007/978-1-4614-9233-7-21

[B20] Chanwitheesuk A. Teerawutgulrag A. Kilburn J. D. Rakariyatham N. (2007). Antimicrobial gallic acid from *Caesalpinia mimosoides* Lamk. Food Chem. x. 100, 1044–1048. 10.1016/j.foodchem.2005.11.008

[B21] Chanwitheesuk A. Teerawutgulrag A. Rakariyatham N. (2005). Screening of antioxidant activity and antioxidant compounds of some edible plants of Thailand. Food Chem. x. 92, 491–497. 10.1016/j.foodchem.2004.07.035

[B22] Charan J. Kantharia N. D. (2013). How to calculate sample size in animal studies? J. Pharmacol. Pharmacother. 4, 303–306. 10.4103/0976-500X.119726 24250214 PMC3826013

[B23] Cheng P. H. (1963). An improved method for the determination of hexosamine in rat skin. J. Invest. Dermatol. 51, 484–490. 10.1038/jid.1968.159 5748733

[B24] Cragg G. M. Newman D. J. (2013). Natural products: A continuing source of novel drug leads. Biochim. Biophys. Acta 1830, 3670–3695. 10.1016/j.bbagen.2013.02.008 23428572 PMC3672862

[B25] Daduang J. Vichitphan S. Daduang S. Hongsprabhas P. Boonsiri P. (2011). High phenolics and antioxidants of some tropical vegetables related to antibacterial and anticancer activities. Afr. J. Pharm. Pharmacol. 5, 608–615. 10.5897/AJPP10.243

[B26] Das S. Bala A. Bhowmik M. Ghosh L. K. (2012). Attenuation of reactive nitrogen species by different flavonoids enriched fractions of Schima Wallichii. Asian pac. J. Trop. Biomed. 2, S632–S636. 10.1016/S2221-1691(12)60287-1

[B27] Dunnill C. Patton T. Brennan J. Barrett J. Dryden M. Cooke J. (2017). Reactive oxygen species (ROS) and wound healing: The functional role of ROS and emerging ROS-modulating technologies for augmentation of the healing process. Int. Wound J. 14, 89–96. 10.1111/iwj.12557 26688157 PMC7950185

[B28] Dwivedi D. Dwivedi M. Malviya S. Singh V. (2016). Evaluation of wound healing, anti-microbial and antioxidant potential of Pongamia pinnata in wistar rats. J. Tradit. Complement. Med. 7, 79–85. 10.1016/j.jtcme.2015.12.002 28053891 PMC5198820

[B29] Działo M. Mierziak J. Korzun U. Preisner M. Szopa J. Kulma A. (2016). The potential of plant phenolics in prevention and therapy of skin disorders. Int. J. Mol. Sci. 17, 160. 10.3390/ijms17020160 26901191 PMC4783894

[B30] Elloumi W. Mahmoudi A. Ortiz S. Boutefnouchet S. Chamkha M. Sayadi S. (2022). Wound healing potential of quercetin-3-O-rhamnoside and myricetin-3-O-rhamnoside isolated from Pistacia lentiscus distilled leaves in rats model. Biomed. Pharmacother. 146, 112574. 10.1016/j.biopha.2021.112574 35062055

[B31] Fikru A. Makonnen E. Eguale T. Debella A. Mekonnen G. A. (2012). Evaluation of *in vivo* wound healing activity of methanol extract of *Achyranthes aspera* L. J. Ethnopharmacol. 143, 469–474. 10.1016/j.jep.2012.06.049 22771316

[B32] Ghane S. G. Attar U. A. Yadav P. B. Lekhak M. M. (2018). Antioxidant, anti-diabetic, acetylcholinesterase inhibitory potential and estimation of alkaloids (lycorine and galanthamine) from Crinum species: An important source of anticancer and anti-Alzheimer drug. Ind. Crops Prod. 125, 168–177. 10.1016/j.indcrop.2018.08.087

[B33] Guimarães I. Baptista-Silva S. Pintado M. Oliveira A. L. (2021). Polyphenols: A promising avenue in therapeutic solutions for wound care. Appl. Sci. (Basel). 11, 1230. 10.3390/app11031230

[B34] Gurung S. Skalko-Basnet N. (2009). Wound healing properties of carica papaya latex: *In vivo* evaluation in mice burn model. J. Ethnopharmacol. 121, 338–341. 10.1016/j.jep.2008.10.030 19041705

[B35] Hadwan M. H. Ali S. K. (2018). New spectrophotometric assay for assessments of catalase activity in biological samples. Anal. Biochem. 542, 29–33. 10.1016/j.ab.2017.11.013 29175424

[B36] Hashemnia M. Nikousefat Z. Mohammadalipour A. Zangeneh M. Zangeneh A. (2019). Wound healing activity of Pimpinella anisum methanolic extract in streptozotocin-induced diabetic rats. J. Wound Care 28, S26–S36. 10.12968/jowc.2019.28.sup10.s26 31600102

[B37] Hazra B. Biswas S. Mandal N. (2008). Antioxidant and free radical scavenging activity of Spondias pinnata. BMC Complement. Altern. Med. 8, 63. 10.1186/1472-6882-8-63 19068130 PMC2636748

[B38] Houghton P. J. Hylands P. J. Mensah A. Y. Hensel A. Deters A. M. (2005). *In vitro* tests and ethnopharmacological investigations: Wound healing as an example. J. Ethnopharmacol. 100, 100–107. 10.1016/j.jep.2005.07.001 16040217

[B4] ICH guidelines (2005). International conference on harmonisation of technical requirements for registration of pharmaceuticals for human use, ICH harmonised tripartite guideline, Validation of analytical procedures: text and methodology Q2(R1). Available at: https://www.gmp-compliance.org/files/guidemgr/Q2(R1).pdf (Accessed January 5, 2022).

[B39] Kakkar P. Das B. Vishwanathan P. N. (1984). A modified spectrophotometric assay of superoxide dismutase. Indian J. biochem. Biophys. 21, 130–132. 6490072

[B40] Lee K. H. (1968). Studies on the mechanism of action of salicylate. II. Retardation of wound healing by aspirin. J. Pharm. Sci. 57, 1042–1043. 10.1002/jps.2600570633 5671327

[B41] Mekonnen A. Sidamo T. Asres K. Engidawork E. (2013). *In vivo* wound healing activity and phytochemical screening of the crude extract and various fractions of Kalanchoe petitiana A. Rich (Crassulaceae) leaves in mice. J. Ethnopharmacol. 145, 638–646. 10.1016/j.jep.2012.12.002 23228912

[B42] Misic A. M. Gardner S. E. Grice E. A. (2014). The wound microbiome: Modern approaches to examining the role of microorganisms in impaired chronic wound healing. Adv. Wound Care 3, 502–510. 10.1089/wound.2012.0397 PMC4086514 25032070

[B43] Moghadam S. E. Ebrahimi S. N. Salehi P. Farimani M. M. Hamburger M. Jabbarzadeh E. (2017). Wound healing potential of chlorogenic acid and myricetin-3-O-β-rhamnoside isolated from Parrotia persica. Molecules 22, E1501. 10.3390/molecules22091501 28885580 PMC5603238

[B44] Moghadamtousi S. Z. Rouhollahi E. Hajrezaie M. Karimian H. Abdulla M. A. Kadir H. A. (2015). Annona muricata leaves accelerate wound healing in rats via involvement of Hsp70 and antioxidant defence. Int. J. Surg. 18, 110–117. 10.1016/j.ijsu.2015.03.026 25899210

[B45] Moron M. S. Depierre J. W. Mannervik B. (1979). Levels of glutathione, glutathione reductase and glutathione S-transferase activities in rat lung and liver. Biochim. Biophys. Acta 582, 67–78. 10.1016/0304-4165(79)90289-7 760819

[B46] Murthy S. Gautam M. K. Goel S. Purohit V. Sharma H. Goel R. K. (2013). Evaluation of *in vivo* wound healing activity of Bacopa monniera on different wound model in rats. Biomed. Res. Int. 2013, 972028. 10.1155/2013/972028 23984424 PMC3745907

[B47] Nayar T. S. Beegam A. R. Sibi M. (2014). Flowering plants of the western Ghats, 1. Palode, Thiruvananthpuram, Kerala: JNTBGRI.

[B48] Nussbaum S. R. Carter M. J. Fife C. E. Vanzo J. D. Haught R. Nusgart M. (2018). An economic evaluation of the impact, cost, and medicare policy implications of chronic non healing wounds. Value Health 21, 27–32. 10.1016/j.jval.2017.07.007 29304937

[B49] OECD (2017). OECD guidelines for the testing of chemicals, acute dermal toxicity: Fixed dose procedure. Paris: OECD Publishing. 10.1787/9789264070585-en

[B50] Oyedepo O. O. Femurewa A. J. (1995). Anti-protease and membrane stabilizing activities of extracts of Fagra zanthoxiloides, Olax subscorpioides and Tetrapleura tetraptera. Int. J. Pharmacogn. 33, 65–69. 10.3109/13880209509088150

[B51] Palasap A. Limpaiboon T. Boonsiri P. Thapphasaraphong S. Daduang S. Suwannalert P. (2014). Cytotoxic effects of Phytophenolics from *Caesalpinia mimosoides* Lamk on cervical carcinoma cell lines through an apoptotic pathway. Asian pac. J. Cancer Prev. 15, 449–454. 10.7314/apjcp.2014.15.1.449 24528072

[B52] Pandey K. B. Rizvi S. I. (2009). Plant polyphenols as dietary antioxidants in human health and disease. Oxid. Med. Cell. Longev. 2, 270–278. 10.4161/oxim.2.5.9498 20716914 PMC2835915

[B53] Perianayagam B. Sharma S. K. Pillai K. K. Pandurangan A. Kesavan D. (2012). Evaluation of antimicrobial activity of ethanol extract and compounds isolated from Trichodesma indicum (Linn.) R. Br. root. J. Ethnopharmacol. 142, 283–286. 10.1016/j.jep.2012.04.020 22543169

[B54] Ponrasu T. Jamuna S. Mathew A. Madhukumar K. N. Ganeshkumar M. Iyappan K. (2013). Efficacy of L-proline administration on the early responses during cutaneous wound healing in rats. Amino acids 45, 179–189. 10.1007/s00726-013-1486-0 23508578

[B55] Ruiz-Ruiz J. C. Matus-Basto A. J. Acereto-Escoffié P. Segura-Campos M. R. (2017). Antioxidant and anti-inflammatory activities of phenolic compounds isolated from Melipona beecheii honey. Food Agric. Immunol. 28, 1424–1437. 10.1080/09540105.2017.1347148

[B56] Schultz G. S. (1999). “Molecular regulations of wound healing,” in Acute and chronic wounds: Nursing management. Editor Bryant R. A.. Second edition (USA: WB Saunders Publisher), 413–429.

[B57] Semwal D. K. Chauhan A. Kumar A. Aswal S. Semwal R. B. Kumar A. (2019). Status of Indian medicinal plants in the international union for conservation of nature and the future of ayurvedic drugs: shouldn’t think about ayurvedic fundamentals? J. Integr. Med. 17, 238–243. 10.1016/j.joim.2019.04.008 31076374

[B58] Shao Y. Dang M. Lin Y. Xue F. (2019). Evaluation of wound healing activity of plumbagin in diabetic rats. Life Sci. 231, 116422. 10.1016/j.lfs.2019.04.048 31059689

[B59] Silja V. P. Varma S. K. Mohanan K. V. (2008). Ethnomedicinal plant knowledge of Mullu kuruma tribe of Waynad district, Kerala. Indian J. Tradit. Know 7, 604–612.

[B60] Silva S. M. M. Costa C. R. R. Gelfuso G. M. Guerra E. N. S. Nóbrega Y. K. M. Gomes S. M. (2019). Wound healing effect of essential oil extracted from Eugenia dysenterica DC (Myrtaceae) leaves. Molecules 24, E2. 10.3390/molecules24010002 PMC6337431 30577426

[B61] Strodtbeck F. (2001). Physiology of wound healing. Newborn Infant Nurs. Rev. 1, 43–52. 10.1053/nbin.2001.23176

[B62] Suntar I. Akkol E. K. Keles H. Yesilada E. Sarker S. D. (2013). Exploration of the wound healing potential of Helichrysum graveolens (Bieb.) Sweet: Isolation of apigenin as an active component. J. Ethnopharmacol. 149, 103–110. 10.1016/j.jep.2013.06.006 23764736

[B63] Suntar I. Koca U. Keles H. Akkol E. K. (2011). Wound healing activity of Rubus sanctus Schreber (Rosaceae): Preclinical study in animal models. Evid. Based. Complement. Altern. Med. 2011, 816156. 10.1093/ecam/nep137 PMC3139958 19755505

[B64] Suntar I. P. Akkol E. K. Yalcin F. N. Koca U. Keles H. Yesilada E. (2010). Wound healing potential of Sambucus ebulus L. leaves and isolation of an active component, quercetin 3-O-glucoside. J. Ethnopharmacol. 129, 106–114. 10.1016/j.jep.2010.01.051 20132876

[B65] Tangsaengvit N. Kitphati W. Tadtong S. Bunyapraphatsara N. Nukoolkarn V. (2013). Neurite outgrowth and neuroprotective effects of quercetin from *Caesalpinia mimosoides* Lamk. on cultured P19-derived neurons. Evid. Based. Complement. Altern. Med. 2013, 838051. 10.1155/2013/838051 PMC3693115 23840266

[B66] Tohidi B. Rahimmalek M. Arzani A. (2017). Essential oil composition, total phenolic, flavonoid contents, and antioxidant activity of Thymus species collected from different regions of Iran. Food Chem. 220, 153–161. 10.1016/j.foodchem.2016.09.203 27855883

[B67] Vittorazzi C. Endringer D. C. de Andrade T. U. Scherer R. Fronza M. (2016). Antioxidant, antimicrobial and wound healing properties of Struthanthus vulgaris. Pharm. Biol. 54, 331–337. 10.3109/13880209.2015.1040515 25915104

[B68] Weckesser S. Engel K. Simon-Haarhaus B. Wittmer A. Pelz K. Schempp C. M. (2007). Screening of plant extracts for antimicrobial activity against bacteria and yeasts with dermatological relevance. Phytomedicine 14, 508–516. 10.1016/j.phymed.2006.12.013 17291738

[B69] Xavier-Santos J. B. Passos J. G. R. Gomes J. A. S. Cruz J. V. C. Alves J. S. F. Garciada Silva V. B. R. M. (2022). Topical gel containing phenolic-rich extract from Ipomoea pes-capre leaf (Convolvulaceae) has anti-inflammatory, wound healing, and antiophidic properties. Biomed. Pharmacother. 149, 112921. 10.1016/j.biopha.2022.112921 36068780

[B70] Yadav E. Singh D. Yadav P. Verma A. (2018). Antioxidant and anti-inflammatory properties of Prosopis cineraria based phenolic rich ointment in wound healing. Biomed. Pharmacother. 108, 1572–1583. 10.1016/j.biopha.2018.09.180 30372859

[B71] Yadav E. Singh D. Yadav P. Verma A. (2017). Attenuation of dermal wounds via downregulating oxidative stress and inflammatory markers by protocatechuic acid rich n-butanol fraction of Trianthema portulacastrum Linn. in wistar albino rats. Biomed. Pharmacother. 96, 86–97. 10.1016/j.biopha.2017.09.125 28965012

[B72] Yodsaoue O. Karalai C. Ponglimanont C. Tewtrakul S. Chantrapromma S. (2010). Potential anti-inflammatory diterpenoids from the roots of *Caesalpinia mimosoides* Lamk. Phytochemistry 71, 1756–1764. 10.1016/j.phytochem.2010.06.016 20656305

