# Supplementary material for: Attenuation of dermal wounds through topical application of ointment containing phenol enriched fraction of Caesalpinia mimosoides Lam
Source: Front Pharmacol. 2022 Oct 13;13:1025848. doi: 10.3389/fphar.2022.1025848 (PMC9608657; doi:10.3389/fphar.2022.1025848)
Supplement: Supplementary file 2 [file Image2.pdf]

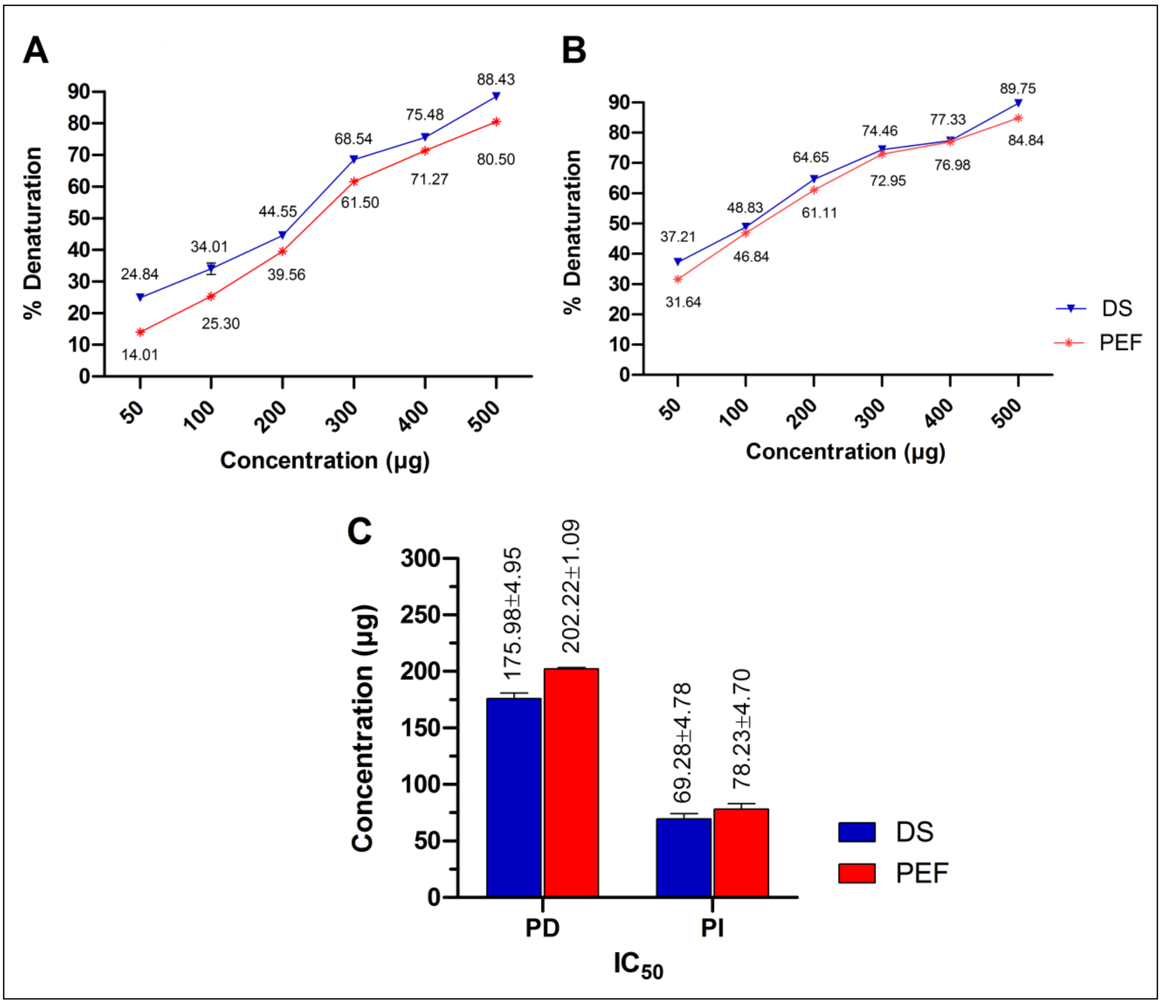

SUPPLEMENTARY FIGURE S2

Anti-inflammatory activity of PEF and standard diclofenac sodium. **(A)** Percentage inhibition of PEF and standard diclofenac sodium in protein denaturation inhibition assay, **(B)** Percentage inhibition of PEF and standard diclofenac sodium in proteinase inhibitory activity, **(C)** IC_{50} values of PEF and standard diclofenac sodium. PD: Protein denaturation inhibitory assay. PI: Proteinase inhibitory activity.
