# Supplementary material for: Attenuation of dermal wounds through topical application of ointment containing phenol enriched fraction of Caesalpinia mimosoides Lam
Source: Front Pharmacol. 2022 Oct 13;13:1025848. doi: 10.3389/fphar.2022.1025848 (PMC9608657; doi:10.3389/fphar.2022.1025848)
Supplement: Supplementary file 3 [file Image3.pdf]

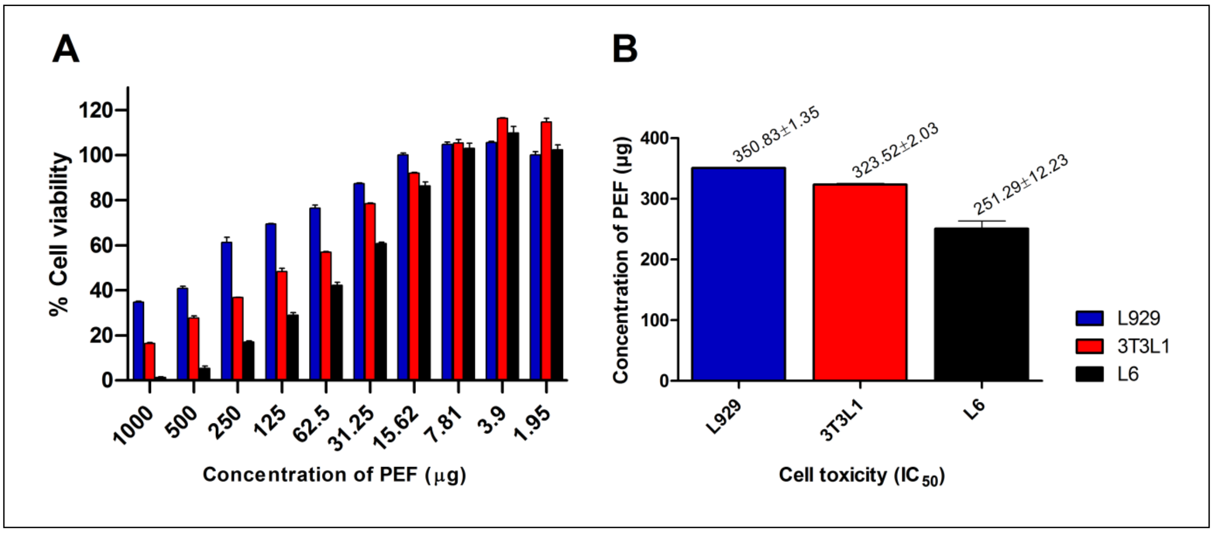

SUPPLEMENTARY FIGURE S3

Viability/toxicity of cell lines. **(A)** Percentage viability of L929, 3T3L1 and L6 cells, **(B)** Cell toxicity (IC_{50}) of corresponding cells.
