# Supplementary material for: Attenuation of dermal wounds through topical application of ointment containing phenol enriched fraction of Caesalpinia mimosoides Lam
Source: Front Pharmacol. 2022 Oct 13;13:1025848. doi: 10.3389/fphar.2022.1025848 (PMC9608657; doi:10.3389/fphar.2022.1025848)
Supplement: Supplementary file 4 [file Image1.pdf]

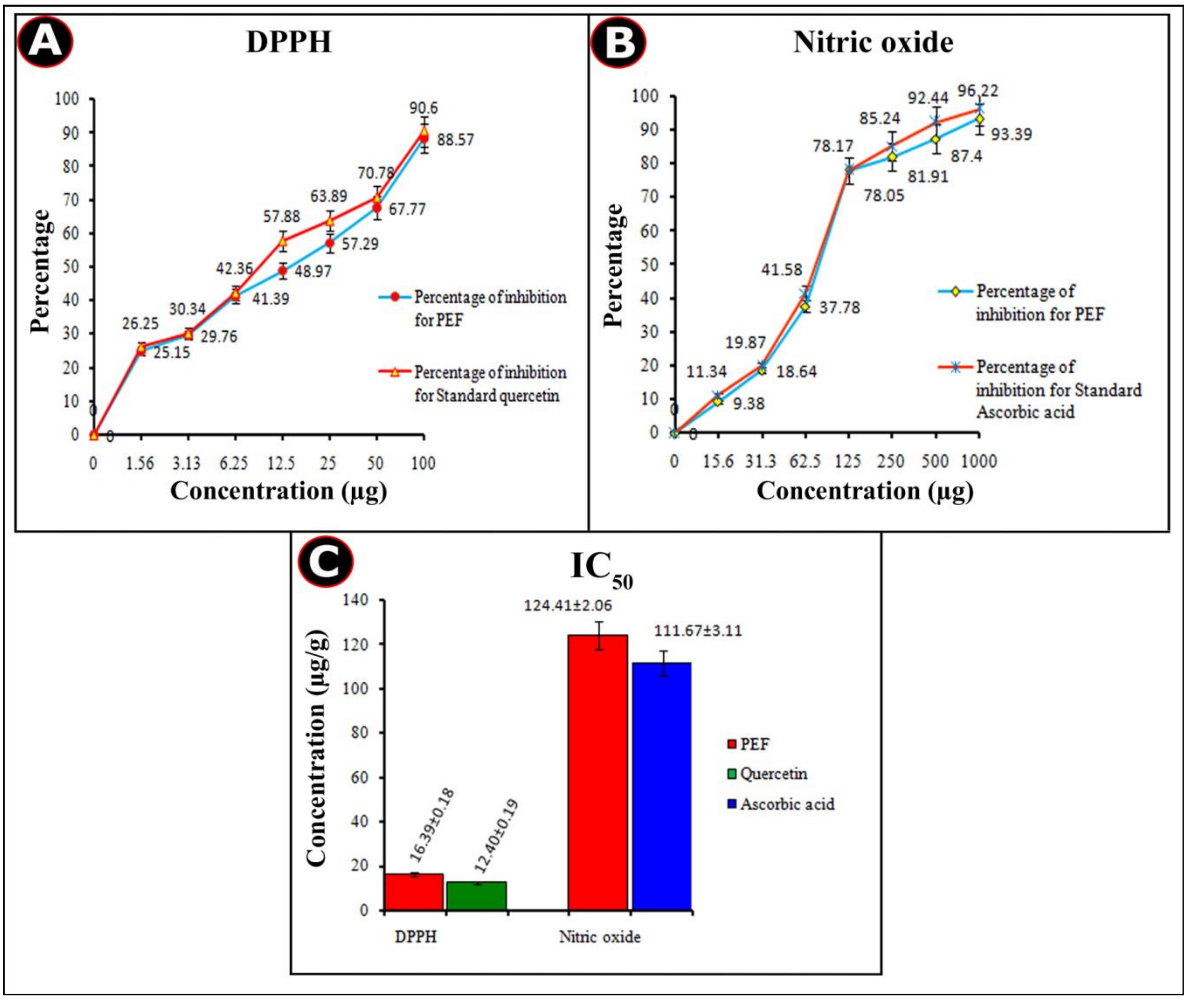

SUPPLEMENTARY FIGURE S1

Antioxidant activity of PEF and corresponding standards. **(A)** Percentage inhibition exhibited by standard quercetin and PEF in DPPH radical scavenging assay, **(B)** Percentage inhibition of standard ascorbic acid and PEF in Nitric oxide radical scavenging assay, **(C)** IC_{50} of PEF and respective standards of antioxidant parameters.
