# Supplementary material for: Attenuation of dermal wounds through topical application of ointment containing phenol enriched fraction of Caesalpinia mimosoides Lam
Source: Front Pharmacol. 2022 Oct 13;13:1025848. doi: 10.3389/fphar.2022.1025848 (PMC9608657; doi:10.3389/fphar.2022.1025848)
Supplement: Supplementary file 1 [file Image4.pdf]

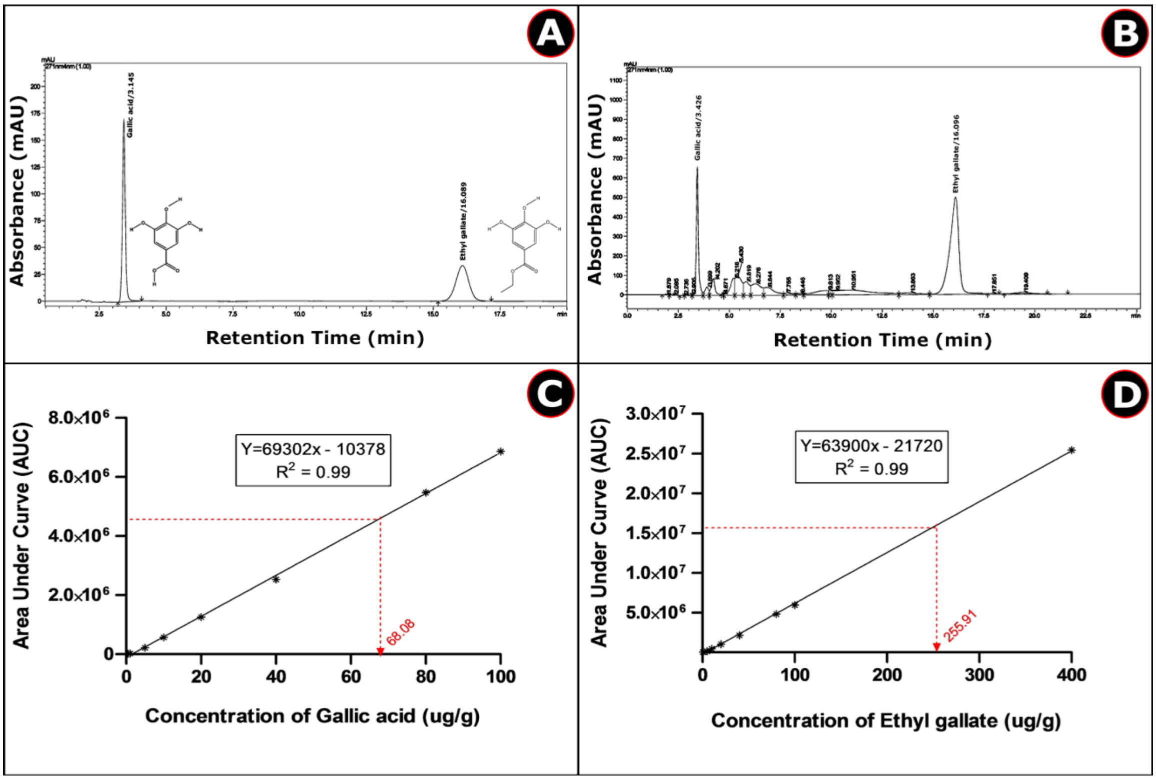

SUPPLEMENTARY FIGURE S4

RP-UFLC-DAD analysis of PEF. **(A)** Chromatograms of standard gallic acid and ethyl gallate, **(B)** Chromatogram of PEF, **(C and D)** Eight point calibration curve equation of standard gallic acid and ethyl gallate.
